# Nonparametric Inference of Conditional Expectile Functions in Large-Scale Time Series Data With Improved Efficiency

**DOI:** 10.1111/jtsa.70021

**Published:** 2025-10-08

**Authors:** Feipeng Zhang, Ping-Shou Zhong

**Affiliations:** 1 School of Economics and Finance, Xi’an Jiaotong University, Xi’an, China; 2 Department of Mathematics, Statistics, and Computer Science, University of Illinois Chicago, Chicago, Illinois, USA

**Keywords:** asymmetric least squares, big data, coherent risk measure, expectiles, strong mixing process, 62M10, 62G08, 62P20

## Abstract

Expectile is a coherent and elicitable law-invariant risk measure widely applied in risk management. Existing methods based on iteratively reweighted least squares (IWLS) are not computationally efficient for large-scale sample sizes. To overcome the issue, we develop a direct nonparametric conditional expectile function estimator by inverting the local polynomial estimator of the conditional loss-gain function. The proposed estimator is computationally friendly and stable without using iterative algorithms that require computation with large-scale data in each iteration. We establish the asymptotic distribution of the proposed estimator. We further show that the proposed estimator has a smaller variance than the existing IWLS estimator and a smaller mean square error in various scenarios. Simulations confirm the computational and statistical efficiency of the proposed method. We further apply the proposed methods to an S&P500 data set to illustrate the computational time to estimate the conditional expectile-based value-at-risk (EVaR) and the precision in out-of-sample prediction.

## 1 | Introduction

Expectile has received substantial attention in statistical finance because it is the only risk measure that satisfies coherence (translation invariance, monotonicity, positive homogeneity and subadditivity, (Artzner et al. 1999)) and elicitablilty (being a minimizer of a suitable expected score, (Bellini and Bignozzi 2015; Ziegel 2016)). Expectiles generalize the concept of expectations and it characterizes the distribution in a way similar to quantiles. The expectile of a cumulative distribution function can be interpreted as the quantile of another distribution related by an explicit formula to the distribution function (Jones 1994). In contrary to quantiles only depending on the frequency of tail realizations, expectiles depend on both tail realizations and their probability (Kuan et al. 2009). Besides, quantiles only use the information on positions of observations, whereas expectiles use the distance to observations (Sobotka and Kneib 2012).

Expectile regression, firstly proposed by Newey and Powell (1987), generalizes conditional mean regression and models the expectation of a response conditional on its being in the tail of the distribution. It has become a popular alternative to quantile regression (Koenker and Bassett 1978) in risk management applications. While quantile regression is more robust to outliers due to the asymmetric $L_{1}$ loss function, expectile regression has several advantages over quantile regression (Sobotka and Kneib 2012). First, expectiles are always strictly increasing, continuous and uniquely defined for any conditional distribution with a finite mean. However, quantile regression is not well-defined for discrete error distributions due to the uniqueness of the quantile. Second, expectile regression is computationally easier than quantile regression, because it employs a smoothly differentiable loss function. Third, the theoretical analysis for expectile regression is more tractable because its asymptotic covariance matrix does not require estimating the density function of the error.

Most existing literature on expectile regression has been focused on parametric expectile regression models, including linear expectile regression models (Newey and Powell 1987; Efron 1991; Taylor 2008; Kuan et al. 2009), and nonlinear expectile regression models (Kim and Lee 2016; Zhang and Li 2017; Zhang and Tu 2025). However, parametric expectile regression assumes a prior linear or nonlinear parametric functional form, which may suffer from model misspecification. As a parallel development to the nonparametric quantile regression methods (e.g., local polynomial estimators of quantile regression in Xu (2013, 2016)), nonparametric expectile regression, which is the focus of the current paper, has recently gained substantial attention in the literature. Yao and Tong (1996) proposed a local linear estimator for nonparametric expectile regression for ρ-mixing data with a univariate covariate, while Adam and Gijbels (2022) proposed a local polynomial estimator for a univariate expectile regression for independent and identically distributed (IID) data. De Rossi and Harvey (2009) and Schnabel and Eilers (2009) studied the nonparametric expectile regression using splines. Yang and Zou (2015) and Yang et al. (2018) proposed a fully nonparametric multiple expectile regression by the gradient tree boosting algorithm and the reproducing kernel Hilbert spaces, respectively. Readers may refer to Adam and Gijbels (2022) and Zhang et al. (2023) for more references. Nonetheless, all the above-mentioned nonparametric expectile estimators are obtained by directly minimizing the expectile-loss function. Since there is no closed-form solution for such a minimization problem, a numerical iterative algorithm is required to obtain the solution. One common algorithm is the iteratively reweighted least squares (IWLS) method. However, this algorithm could be unstable and computationally expensive, especially for large-scale data sets.

This paper is motivated by the practical needs in estimating and inferring expectile functions for large-scale time series data. Large-scale time series data are frequently encountered in various applications with the availability of modern techniques for recording and storage. One motivating example in this paper is the daily S&P500 stock index prices from January 2, 1980 to November 30, 2022, which includes a total of 10,825 observations. Based on our numerical experiments, it can take hours to compute the estimation of the nonparametric expectile functions using the existing IWLS algorithm. Please see Section 4.2 for more details.

To overcome the computational challenges, we develop a computationally efficient local polynomial method to estimate a nonparametric conditional expectile function for time series data. The main idea is to construct a closed-form estimator by representing the expectile function as an inverse function of a conditional loss-gain ratio function. Basically, the loss-gain ratio function is the reciprocal of the conditional Omega ratio function developed by Keating and Shadwick (2002). Due to the strict monotone decreasing property of the Omega ratio function (Bellini et al. 2018), the inverse of the conditional loss-gain function is well-defined. To estimate the conditional loss-gain ratio function, we apply the local polynomial estimator. First and most importantly, unlike many existing nonparametric expectile estimators obtained numerically as a minimizer of the expectile-loss function, the proposed estimator is computationally simple and efficient with tractable and rigorous theoretical properties. Extensive simulation studies and an empirical application illustrate that our proposed estimator outperforms the existing method in finite-sample performance and computational efficiency.

In addition to computational advantages, we formally prove that the proposed estimator is asymptotically more efficient than the existing IWLS nonparametric estimator when the local polynomial method with order p=0 is applied or they are compared at τ=0.5 conditional expectile for p>0, in the sense that the asymptotic mean squared error (MSE) of our proposed estimator is always less than that of the IWLS nonparametric estimator. If undersmoothing is applied, the proposed estimator is always asymptotically more efficient than the IWLS estimator. As a result, a Wald-type confidence interval based on the proposed estimator is narrower than that based on the IWLS estimators.

Our work is complementary to the kernel-type estimator of the expectile regression studied by Mohammedi et al. (2021) and Almanjahie et al. (2022) for functional predictors. Our estimator is based on the local polynomial estimator of order p, which may be considered as a generalization of their methods only for IID data. However, their estimators are designed for a functional predictor and a scalar response, while our estimator is for time series data. Apparently, their asymptotic results for functional data cannot be applied to time series data. Second, they did not investigate large-scale time series data, which is a focus of the paper. Third, there are no formal studies comparing the theoretical efficiency between the existing IWLS estimators and the closed-form direct estimators based on the loss-gain ratio function.

The remainder of this paper is structured as follows. Section 2 introduces the proposed methodology in detail. A new and direct local polynomial estimator for the conditional expectile regression is proposed, and an asymptotic distribution for the proposed estimator is developed for stationary α-mixing time series. Section 3 discusses the implementation issues, including the variance estimation and the selection of bandwidth. In Section 4, Monte Carlo simulation studies are conducted to evaluate the finite sample performance of the proposed estimator, and it is also used in an empirical application to the conditional EVaR of S&P500 returns. The extension to the case of multiple covariates is discussed in Section 5. Section 6 concludes the paper. All technical proofs of the main theoretical results are presented in the Supporting Information.

## 2 | Nonparametric Expectile Estimators: Existing and Proposed Methods

Consider a two-dimensional stationary time series $\left\{\left(Y_{t},X_{t}\right)\;:\;t=\right.1,\ldots ,n\}$, where $Y_{t}$ is the dependent variable of interest and $X_{t}$ is the covariate. To fix the idea and speed up the exploration, we assume $X_{t}$ is a univariate covariate. An extension to the general case with multivariate covariates with both discrete and continuous variables will be discussed in Section 5.

For τ∈(0,1), the τth conditional expectile of $Y_{t}$ given $X_{t}=x,\mu_{\tau }(x)$, is defined as the minimizer of an asymmetric loss function (Newey and Powell 1987):

$$\mu_{\tau }(x)=\text{arg}\;\underset{y\in R}{\text{min}}\;\text{E}\left[\Psi_{\tau }\left(Y_{t}-y\right)|X_{t}=x\right],$$

where $\Psi_{\tau }(u)=|\tau -I(u\leq 0)|u^{2}≕w_{\tau }(u)u^{2}$ is the asymmetric squared error loss function, and I(⋅) is the indicator function. Obviously, when $\tau =0.5,\mu_{\tau }(x)$ reduces to the conditional mean $\text{E}\left[Y_{t}|X_{t}=x\right]$. In this sense, expectile regression could be viewed as a generalization of conditional mean regression. In fact, a τ-expectile $\mu_{\tau }$ of a distribution F(⋅) may be understood as the mean of F(⋅) if values above $\mu_{\tau }$ occurred τ/(1-τ) as often as they do (Philipps 2022). An asymmetric loss function is of interest in many applications. For example, to estimate insurance premiums, insurance companies would rather overestimate costs rather than underestimate them to remain profitable. Please also refer to Newey and Powell (1987); Kuan et al. (2009); Philipps (2022) for more discussions.

### 2.1 | Existing IWLS Estimators

In this subsection, we will review the existing IWLS estimators based on minimizing expectile loss function for stationary time series. The local polynomial estimator for the nonparametric expectile function (Yao and Tong 1996; Adam and Gijbels 2022) is defined as ${\tilde{\mu }}_{\tau }(x)=e_{1}^{\text{T}}\tilde{\beta }$, where $e_{1}=(1,0,\ldots ,0{)}^{\text{T}}$ is a (p+1)-dimensional vector, $\tilde{\beta }={\left({\tilde{\beta }}_{0},\ldots ,{\tilde{\beta }}_{p}\right)}^{⊤}$ is obtained by

(1) $$\tilde{\beta }=\text{arg}\;\underset{\beta_{0},\ldots ,\beta_{p}}{\text{min}}\sum_{t=1}^{n}\Psi_{\tau }\left(Y_{t}-\sum_{j=0}^{p}\beta_{j}{\left(X_{t}-x\right)}^{j}\right)K_{h}\left(X_{t}-x\right),$$

$K_{h}(u)=K(u/h)/h$, and K(⋅) is a kernel function with a smoothing bandwidth h>0. It is not difficult to show that $X^{⊤}W(\beta )X\beta =X^{⊤}W(\beta )Y$, where

$$X=\left[\begin{array}{cccc} 1 & \left(X_{1}-x\right) & \ldots & {\left(X_{1}-x\right)}^{p}\\ \vdots & \vdots & \ddots & \vdots \\ 1 & \left(X_{n}-x\right) & \ldots & {\left(X_{n}-x\right)}^{p} \end{array}\right],\;Y=\left[\begin{array}{c} Y_{1}\\ \vdots \\ Y_{n} \end{array}\right],$$

$$W(\beta )=\left[\begin{array}{ccc} w_{1}(\tau ,\beta )K_{h}\left(X_{1}-x\right) & \ldots & 0\\ \vdots & \ddots & \vdots \\ 0 & \ldots & w_{n}(\tau ,\beta )K_{h}\left(X_{n}-x\right) \end{array}\right],$$

where $w_{t}(\tau ,\beta )=\left|\tau -I\left\{Y_{t}\leq \sum_{j=0}^{p}\beta_{j}{\left(X_{t}-x\right)}^{j}\right\}\right|$ and $\beta ={\left(\beta_{0},\ldots ,\beta_{p}\right)}^{⊤}$. Because W(β) depends on β, it requires an iterative numerical algorithm to solve β. We summarize the iterative reweighted least squares (IWLS) in the following Algorithm 1.

**Algorithm 1.** The iterative reweighted least squares (IWLS) procedure.

1. Initialize ${\tilde{\beta }}^{\left(0\right)}$ by the ordinary (or local linear) least squares estimator.
2. Updating ${\tilde{\beta }}^{\left(k+1\right)}$ by ${\tilde{\beta }}^{\left(k+1\right)}={\left[X^{⊤}W\left({\tilde{\beta }}^{\left(k\right)}\right)X\right]}^{-1}X^{⊤}W\left({\tilde{\beta }}^{\left(k\right)}\right)Y$ for k=0,1,….
3. Repeat the iteration in Step 2 until convergence with some stopping criterion, for example, $\left\|{\tilde{\beta }}^{\left(k+1\right)}-{\tilde{\beta }}^{\left(k\right)}\right\|<10^{-6}$.

Adam and Gijbels (2022) discussed the convergence of the IWLS algorithm, by referring to a convergence analysis with convex criterion functions (Wolke and Schwetlick 1988). The authors also asserted that the value of ${\tilde{\beta }}^{\left(k+1\right)}$ after convergence is exactly the minimizer of Equation (1), because the objective function (1) is convex due to the convexity of the expectile loss function.

For large-scale time series with large n, the computation complexity of computing every element in the matrix $X^{⊤}W(\beta )X$ is at the order of n. Because the matrix $X^{⊤}W(\beta )X$ depends on β, in each iteration of the IWLS algorithm, it requires to compute the matrix repeatedly (see Step 2 of the Algorithm 1). Moreover, the nonparametric estimator depends on the tuning parameter bandwidth h. For every candidate bandwidth h, one needs to compute the IWLS estimator to minimize some certain criterion (specified in Section 3.2). Thus, it is time-consuming to apply the IWLS algorithm for time series data with large sample size, especially when the number of candidate bandwidths is also large. Please also refer to Section 4.2 for a detailed numerical example.

### 2.2 | A New Estimator

In this subsection, we will introduce our proposed estimator. A key idea of our method is to treat the conditional expectile as a function of conditional means and then to consider it as a generalization of conditional mean. By a simple manipulation, $\mu_{\tau }(x)$ satisfies the following first order condition

$$G\left\{\mu_{\tau }(x)|x\right\}=\frac{\tau }{1-\tau },$$

where

$$G(y|x)=\frac{\text{E}\left[{\left(Y_{t}-y\right)}_{-}|X_{t}=x\right]}{\text{E}\left[{\left(Y_{t}-y\right)}_{+}|X_{t}=x\right]},$$

$a_{-}=\text{max}(-a,0)$ and $a_{+}=\text{max}(a,0)$ are the negative and positive parts of a real number a, respectively. Consequently, conditional expectile can be interpreted as the threshold that provides a ratio between losses and gains by the odds quantity τ/(1-τ). Note that G(y∣x) is identical to the reciprocal of the conditional Omega ratio, which is proposed by Keating and Shadwick (2002). More precisely, the Omega ratio or gain-loss ratio is the ratio of the expected gain to the expected loss,

$$\Omega (y|x)=\frac{\text{E}\left[{\left(Y_{t}-y\right)}_{+}|X_{t}=x\right]}{\text{E}\left[{\left(Y_{t}-y\right)}_{-}|X_{t}=x\right]}.$$

For the unconditional version, Bellini et al. (2018) showed that Omega ratio, $\Omega_{Y_{t}}(y)=\text{E}{\left(Y_{t}-y\right)}_{+}/\text{E}{\left(Y_{t}-y\right)}_{-}$ is strictly positive, continuous and strictly decreasing with respect to y. Then, one can readily show that the function G(y∣x) is a strictly increasing function with respect to y given x, see also Mohammedi et al. (2021).^1^ Then, by the monotonicity of G(y∣x), we can write the conditional expectile $\mu_{\tau }(x)$ by

$$\mu_{\tau }(x)=\text{inf}\left\{y\in R\;:\;G(y|x)\geq \frac{\tau }{1-\tau }\right\}.$$

To construct a nonparametric estimator of $\mu_{\tau }(x)$, we first employ local polynomial estimators for $G(y|x)=G_{1}(y|x)/G_{2}(y|x)$, where

$$G_{1}(y|x)≔\text{E}\left[{\left(Y_{t}-y\right)}_{-}|X_{t}=x\right],\;\text{and}$$

$$G_{2}(y|x)≔\text{E}\left[{\left(Y_{t}-y\right)}_{+}|X_{t}=x\right].$$

More specifically, we estimate G(y∣x) by

(2) $$\hat{G}(y|x)=\frac{{\hat{G}}_{1}(y|x)}{{\hat{G}}_{2}(y|x)}≔\frac{n^{-1}\sum_{t=1}^{n}W_{p}\left\{\left(X_{t}-x\right)/h\right\}{\left(Y_{t}-y\right)}_{-}}{n^{-1}\sum_{t=1}^{n}W_{p}\left\{\left(X_{t}-x\right)/h\right\}{\left(Y_{t}-y\right)}_{+}},$$

where the kernel weights $W_{p}(u)=e_{1}^{⊤}H^{-1}S_{n}^{-1}H^{-1}{\left(1,u,\ldots ,u^{p}\right)}^{⊤}K(u)$, with the (p+1)-dimensional vector $e_{1}=(1,0,\ldots ,0{)}^{⊤},H=\text{diag}\left(1,h,\ldots ,h^{p}\right)$, and $S_{n}={\left[S_{n,i+j-2}\right]}_{1\leq i,j\leq p}$ is a (p+1)×(p+1) matrix with $S_{n,j}=n^{-1}\sum_{t=1}^{n}{\left(\frac{X_{t}-x}{h}\right)}^{j}K_{h}\left(X_{t}-x\right)$ for j=0,…,2p.

For simplification, we can write $\hat{G}(y|x)$ in matrix notations,

(3) $$\hat{G}(y|x)=\frac{{\hat{G}}_{1}(y|x)}{{\hat{G}}_{2}(y|x)}≔\frac{e_{1}^{⊤}S_{n}^{-1}H^{-1}X_{n}^{⊤}W_{n}Y_{n}^{-}(y)}{e_{1}^{⊤}S_{n}^{-1}H^{-1}X_{n}^{⊤}W_{n}Y_{n}^{+}(y)},$$

where the matrices $W_{n}=\text{diag}\left\{K_{h}\left(X_{1}-x\right),\ldots ,K_{h}\right.\left.\left(X_{n}-x\right)\right\}$ and

$$X_{n}=\left[\begin{array}{ccc} 1 & \cdots & {\left(\frac{X_{1}-x}{h}\right)}^{p}\\ \vdots & \ddots & \vdots \\ 1 & \cdots & {\left(\frac{X_{n}-x}{h}\right)}^{p} \end{array}\right],$$

$Y_{n}^{-}(y)={\left({\left(Y_{1}-y\right)}_{-},\ldots ,{\left(Y_{n}-y\right)}_{-}\right)}^{⊤}$ and $Y_{n}^{+}(y)=\left({\left(Y_{1}-y\right)}_{+},\ldots \right.,{\left.{\left(Y_{n}-y\right)}_{+}\right)}^{⊤}$. Note that both the numerator and the denominator include $e_{1}^{⊤}S_{n}^{-1}H^{-1}X_{n}^{⊤}W_{n}$, so we only need to compute this weight vector once. Therefore, the conditional expectile $\mu_{\tau }(x)$ can be estimated by

$${\hat{\mu }}_{\tau }(x)=\text{inf}\left\{y\in R\;:\;\hat{G}(y|x)\geq \frac{\tau }{1-\tau }\right\}.$$

To obtain the inverse function of $\hat{G}(y|x)$ for a given τ and x, we first specify an interval $\left(l_{y},u_{y}\right)$ that contains the solution ${\hat{\mu }}_{\tau }(x)$. Let $T_{l}=\left\{t\in \{1,\ldots ,n\}\;:\;Y_{t}<l_{y}\right\}$ be a set of indices of $Y_{t}$ less than $l_{y}$, and $T_{m}=\left\{t\in \{1,\ldots ,n\}\;:\;l_{y}\leq Y_{t}\leq u_{y}\right\}$ be a set of indices of $Y_{t}$ within $\left[l_{y},u_{y}\right]$. Let $l_{y}\leq Y_{k_{1}}\leq \cdots \leq Y_{k_{m}}\leq u_{y}$ be the ordered $Y_{t}$ for $t\in T_{m}$ and $m=\left|T_{m}\right|$. We can compute ${\hat{G}}_{1}(y|x)$ efficiently using an iterative formula. We first compute ${\hat{G}}_{1}\left(l_{y}|x\right)=n^{-1}\sum_{t\in T_{l}}W_{p}\left\{\left(X_{t}-x\right)/h\right\}\left(l_{y}-Y_{t}\right)$ and ${\hat{G}}_{1}\left(Y_{k_{1}}|x\right)={\hat{G}}_{1}\left(l_{y}|x\right)+W_{np}^{*}\left(y_{k_{1}}-l_{y}\right)$ where $W_{np}^{*}=n^{-1}\sum_{t\in T_{t}}W_{p}\left\{\left(X_{t}-x\right)/h\right\}$. Then, for j=2,…,m, we apply an iterative formula $W_{np}^{*}=W_{np}^{*}+W_{p}\left\{\left(X_{k_{\left(j-1\right)}}-x\right)/(nh)\right\}$ and ${\hat{G}}_{1}\left(y_{k_{j}}|x\right)={\hat{G}}_{1}\left(y_{k_{\left(j-1\right)}}|x\right)+W_{np}^{*}\left\{y_{k_{j}}-y_{k_{\left(j-1\right)}}\right\}$. So the computational complexity for computing all the ${\hat{G}}_{1}(y|x)$ at $y=y_{k_{1}},\ldots ,y_{k_{m}}$ simultaneously is of order O(m). Similarly, the numerator ${\hat{G}}_{2}(y|x)$ of $\hat{G}(y|x)$ can be computed simultaneously for $y=y_{k_{1}},\ldots ,y_{k_{m}}$ with a computational complexity at the order of O(m). Because $\hat{G}(y|x)={\hat{G}}_{1}(y|x)/{\hat{G}}_{2}(y|x)$ are computed for all $y=y_{k_{1}},\ldots ,y_{k_{m}}$, the corresponding inverse function ${\hat{G}}^{-1}(\cdot |x)$ can be obtained directly based on $\hat{G}\left(y_{k_{1}}|x\right),\ldots ,\hat{G}\left(y_{k_{m}}|x\right)$ due to its monotonicity. Compared with the IWLS estimator in Section 2.1, our proposed estimator and algorithm do not need to evaluate the estimator that requires computation involving a large-scale data set in each iteration. Therefore, our method is expected to be more computationally efficient than the IWLS estimator. In Section 4, numerical examples illustrate that our proposed estimator outperforms the IWLS estimator in finite-sample performance and computational efficiency.

In the proposed estimator (2), when p=0, both ${\hat{G}}_{1}(y|x)$ and ${\hat{G}}_{2}(y|x)$ are local constant estimators, which was investigated by Mohammedi et al. (2021) for IID data. However, their results can not be applied for time series data. Then, we aim to establish the rigorous asymptotic properties of the proposed estimator for time series data. In general, one could use local polynomial estimators with different orders to estimate $G_{1}(y|x)$ and $G_{2}(y|x)$. For example, we can use local polynomials with orders $p_{1}$ and $p_{2}$ to estimate $G_{1}(y|x)$ and $G_{2}(y|x)$, respectively. Here, $p_{1},p_{2}\geq 1$ could be different integers. To simplify our notations, we focus on $p_{1}=p_{2}=p$ in this paper but it is not difficult to extend our results to a general case.

### 2.3 | Asymptotic Theory

In this section, we establish the consistency and asymptotic normality for the proposed estimator ${\hat{\mu }}_{\tau }(x)$ in Section 2.2 for α-mixing time series. We make the following regularity conditions. Let C be a generic finite constant.

**Assumption A1** The kernel function K(⋅) is symmetric with a bounded support, say [−1, 1].

**Assumption A2** The marginal density $f_{X}(x)$ of $X_{t}$ satisfies $0<f_{X}(x)\leq C<\infty$. The joint density $f_{X_{1},X_{l}}\left(x_{0},x_{l}\right)$ of $X_{1}$ and $X_{l}$ satisfies $0<f_{X_{1},X_{l}},\left(x_{0},x_{l}\right)\leq C<\infty$ for every l≥2.

**Assumption A3** The process $\left\{X_{t},Y_{t}\right\}$ is stationary α-mixing with the mixing coefficients α(l) satisfying $\sum_{l=1}^{\infty }l^{a}\alpha (l{)}^{1-2/\delta }<\infty$ for some δ>2 and a>1-2/δ, and $\text{E}\left[|Y{|}^{\delta }|X=x\right]\leq C<\infty$.

**Assumption A4** (i) The bandwidth h satisfies $h\to 0,nh\to \infty ,nh^{2p+3}=O(1)$, as n→∞; (ii) There exists a sequence of positive integers $s_{n}$ such that $s_{n}\to \infty ,s_{n}=o(\sqrt{nh})$ and $\alpha \left(s_{n}\right)\sqrt{n/h}\to 0$, as n goes to infinity, where α(⋅) is the coefficient function defined in Assumption (A3).

**Assumption A5** (i) Both $G_{1}(y|x)$ and $G_{2}(y|x)$ are (p+1)th continuously differentiable in a neighborhood of y for each fixed x; (ii) The conditional variances $\sigma_{1}^{2}(y|x)=\text{Var}\left({\left(Y_{t}-y\right)}_{-}|X_{t}=x\right)$ and $\sigma_{2}^{2}(y|x)=\text{Var}\left({\left(Y_{t}-y\right)}_{+}|X_{t}=x\right)$ are finite and continuous in y at the point x.

Assumptions A1–A5 are fairly standard for the local polynomial estimator in the time series context, see Masry and Fan (1997), also in Fan and Yao (2003); Xu (2013, 2016). Assumption A1 imposes the symmetric and bounded condition for the kernel function. Assumption A2 requires the bounded condition for the marginal density of $X_{t}$. Assumption A3 imposes moment conditions, and restricts serial dependence of time series, and is reasonably weak. It is equivalent to $\alpha (l)=O\left(1/l^{2+\epsilon }\right)$ for some ϵ>0. Assumption A4 imposes conditions on the bandwidth h which depends on the mixing coefficients. Assumption A5 is required for establishing the asymptotic normality.

Throughout this paper, we use the notation $m^{\left(j\right)}(y|x)=\frac{\partial^{j}}{\partial x^{j}}m(y|x)$ to denote the jth order partial derivative of the function m(y∣x) with respect to x for j≥1, where m(y∣x) is a function of x and y. For the convenience of notation, we denote

$$S=\left[\begin{array}{ccc} \mu_{0} & \cdots & \mu_{p}\\ \vdots & \ddots & \vdots \\ \mu_{p} & \cdots & \mu_{2p} \end{array}\right],\;S^{*}=\left[\begin{array}{ccc} v_{0} & \cdots & v_{p}\\ \vdots & \ddots & \vdots \\ v_{p} & \cdots & v_{2p} \end{array}\right],\;\text{and}\;c_{p}=\left[\begin{array}{c} \mu_{p+1}\\ \vdots \\ \mu_{2p+1} \end{array}\right],$$

where $\mu_{j}=\int u^{j}K(u)du$ and $v_{j}=\int u^{j}K^{2}(u)du$ for j=0,…,2p. We also define $B_{k}(y|x)=G_{k}^{\left(p+1\right)}(y|x)e_{1}^{⊤}S^{-1}c_{p}/(p+1)!$ for k=1,2, and $V_{0}=e_{1}^{⊤}S^{-1}S^{*}S^{-1}e_{1}$.

The following result provides the limiting distribution of $\hat{G}(y|x)$ defined in (2).

**Theorem 1.** *Assume that Assumptions A*1–*A*5 *hold. Then, as* n→∞, *we have*

$$\sqrt{nh}\left[\hat{G}(y|x)-G(y|x)-B_{G}(y|x)\right]\overset{D}{\to }N\left(0,\Sigma_{G}(y|x)\right),$$

*where the bias term and asymptotic variance are*

$$B_{G}(y|x)=\left[\frac{1}{G_{2}(y|x)}B_{1}(y|x)-\frac{G(y|x)}{G_{2}(y|x)}B_{2}(y|x)\right]h^{p+1},$$

$$\Sigma_{G}(y|x)=\frac{V_{0}}{f_{X}(x)}\left[\frac{\sigma_{1}^{2}(y|x)}{G_{2}(y|x{)}^{2}}+\frac{G(y|x{)}^{2}\sigma_{2}^{2}(y|x)}{G_{2}(y|x{)}^{2}}\right]$$

*respectively*.

By Theorem 1, we can establish the asymptotic normality of ${\hat{\mu }}_{\tau }(x)$.

**Theorem 2.** *Assume that Assumptions A1–A5 hold. Then, as* n→∞, *we have*

$$\sqrt{nh}\left[{\hat{\mu }}_{\tau }(x)-\mu_{\tau }(x)-B_{\tau }(x)\right]\overset{D}{\to }N\left(0,\Sigma_{\tau }(x)\right),$$

*where the bias term* $B_{\tau }(x)=-{\left[G^{\prime }\left(\mu_{\tau }(x)|x\right)\right]}^{-1}B_{G}\left(\mu_{\tau }(x)|x\right)$, *and the asymptotic variance* $\Sigma_{\tau }(x)={\left[G^{\prime }\left(\mu_{\tau }(x)|x\right)\right]}^{-2}\Sigma_{G}\left(\mu_{\tau }(x)|x\right)$,

$$G^{\prime }(y|x)=\frac{dG(y|x)}{dy}=\frac{F(y|x)G_{2}(y|x)+(1-F(y|x))G_{1}(y|x)}{G_{2}^{2}(y|x)},$$

*and* F(y∣x) *is the conditional distribution of* $Y_{t}$ given $X_{t}=x$.

Similar to Theorem 2, we can establish the asymptotic properties of ${\tilde{\mu }}_{\tau }(x)$. More specifically,

$$\sqrt{nh}\left[{\tilde{\mu }}_{\tau }(x)-\mu_{\tau }(x)-{\tilde{B}}_{\tau }(x)\right]\overset{D}{\to }N\left(0,{\tilde{\Sigma }}_{\tau }(x)\right),$$

where the bias term and the asymptotic variance are respectively,

$${\tilde{B}}_{\tau }(x)=\frac{\mu_{\tau }^{\left(p+1\right)}(x)e_{1}^{⊤}S^{-1}c_{p}}{(p+1)!}h^{p+1}\;\text{and}\;{\tilde{\Sigma }}_{\tau }(x)=\frac{V_{0}}{f_{X}(x)}\frac{{\tilde{\sigma }}_{\tau }^{2}(x)}{\gamma_{\tau }^{2}(x)},$$

and

$$\gamma_{\tau }(x)=\text{E}\left[w_{\tau }\left(Y_{t}-\mu_{\tau }(x)\right)|X_{t}=x\right]\;=\tau P\left(Y_{t}\leq \mu_{\tau }(x)|X_{t}=x\right)+(1-\tau )P\left(Y_{t}>\mu_{\tau }(x)|X_{t}=x\right),$$

$${\tilde{\sigma }}_{\tau }^{2}(x)=\text{E}\left[{\left[Y_{t}-\mu_{\tau }(x)\right]}^{2}w_{\tau }{\left(Y_{t}-\mu_{\tau }(x)\right)}^{2}|X_{t}=x\right]\;=\int {\left[Y_{t}-\mu_{\tau }(x)\right]}^{2}w_{\tau }{\left(Y_{t}-\mu_{\tau }(x)\right)}^{2}f_{Y|X}(y|x)dy$$

where $f_{Y|X}(y|x)$ is the conditional density function of $Y_{t}$ given $X_{t}=x$.

It is of interest to compare the proposed estimator ${\hat{\mu }}_{\tau }(x)$ and the IWLS estimator ${\tilde{\mu }}_{\tau }(x)$. First, by a simple manipulation, the asymptotic bias of ${\hat{\mu }}_{\tau }(x)$ is exactly

$$B_{\tau }(x)=\frac{\tau G_{2}^{\left(p+1\right)}\left(\mu_{\tau }(x)|x\right)-(1-\tau )G_{1}^{\left(p+1\right)}\left(\mu_{\tau }(x)|x\right)}{\tau \left[1-F\left(\mu_{\tau }(x)|x\right)\right]+(1-\tau )F\left(\mu_{\tau }(x)|x\right)}\frac{e_{1}^{⊤}S^{-1}c_{p}}{(p+1)!}h^{p+1}.$$

It can be shown that $B_{\tau }(x)={\tilde{B}}_{\tau }(x)$ for all τ when p=0, and for τ=0.5 when p=1,2. Detailed proof can be found in the Supporting Information. Furthermore, the following theorem shows that the asymptotic variance of ${\hat{\mu }}_{\tau }(x)$ is always less than that of ${\tilde{\mu }}_{\tau }(x)$.

**Theorem 3.** *Under the assumptions in Theorem 2, we have* $\Sigma_{\tau }(x)\leq {\tilde{\Sigma }}_{\tau }(x)$. *The equality holds if and only if* $Y_{t}$’*s are constants*.

If Wald-type confidence intervals are constructed based on the asymptotic normality of ${\hat{\mu }}_{\tau }(x)$ and ${\tilde{\mu }}_{\tau }(x)$ (see Section 3 for details), Theorem 3 suggests that the average length of the confidence interval for ${\hat{\mu }}_{\tau }(x)$ would be shorter than that of ${\tilde{\mu }}_{\tau }(x)$. This is also confirmed by our simulation studies in Section 4. The results in Theorem 3 together with the comparison between the asymptotic bias terms ${\hat{\mu }}_{\tau }(x)$ and ${\tilde{\mu }}_{\tau }(x)$ imply that the proposed estimator ${\hat{\mu }}_{\tau }(x)$ is asymptotically more efficient (in MSE) than the IWLS estimator ${\tilde{\mu }}_{\tau }(x)$ when p=0 and τ=0.5 when p=1,2. Although the proposed estimator is more efficient than the IWLS estimator, it is not clear if the proposed estimator achieves certain efficiency bound (Bickel et al. 1993). We will investigate this topic as a future research study.

## 3 | Practical Implementation

### 3.1 | Confidence Interval and Variance Estimator

To make statistical inference for ${\hat{\mu }}_{\tau }(x)$, it requires estimating the asymptotic variance $\Sigma_{\tau }(x)$. To this end, we propose the plug-in estimator of $\Sigma_{\tau }(x)$ by

(4) $${\hat{\Sigma }}_{\tau }(x)={\left[{\hat{G}}^{\prime }\left({\hat{\mu }}_{\tau }(x)|x\right)\right]}^{-2}{\hat{\Sigma }}_{G}\left({\hat{\mu }}_{\tau }(x)|x\right),$$

where

$${\hat{\Sigma }}_{G}(y|x)=\frac{V_{0}}{\hat{f}(x)}\left[\frac{{\hat{\sigma }}_{1}^{2}(y|x)}{{\hat{G}}_{2}^{2}(y|x)}+\frac{\hat{G}(y|x{)}^{2}{\hat{\sigma }}_{2}^{2}(y|x)}{{\hat{G}}_{2}^{2}(y|x)}\right],$$

$${\hat{G}}^{\prime }\left({\hat{\mu }}_{\tau }(x)|x\right)=\frac{\hat{F}(y|x){\hat{G}}_{2}(y|x)+(1-\hat{F}(y|x)){\hat{G}}_{1}(y|x)}{{\hat{G}}_{2}^{2}(y|x)},$$

with $\hat{F}(y|x)=n^{-1}\sum_{t=1}^{n}K_{h}\left(X_{t}-x\right)I\left(Y_{t}\leq y\right)/\hat{f}(x)$, and $\hat{f}(x)=n^{-1}\sum_{t=1}^{n}K_{h}\left(X_{t}-x\right)$,

$${\hat{\sigma }}_{1}^{2}\left(y|x\right)=\frac{\frac{1}{n}\sum_{t=1}^{n}K_{h}\left(X_{t}-x\right){\left[{\left(Y_{t}-y\right)}_{-}\right]}^{2}}{\hat{f}\left(x\right)},$$

$${\hat{\sigma }}_{2}^{2}\left(y|x\right)=\frac{\frac{1}{n}\sum_{t=1}^{n}K_{h}\left(X_{t}-x\right){\left[{\left(Y_{t}-y\right)}_{+}\right]}^{2}}{\hat{f}\left(x\right)},$$

and ${\hat{G}}_{1}(y|x)$ and ${\hat{G}}_{2}(y|x)$ are defined in (2). Then, the Wald confidence interval for ${\hat{\mu }}_{\tau }(x)$ at 100(1-α)% significance level is

(5) $$\left[{\hat{\mu }}_{\tau }(x)-z_{1-\alpha /2}\sqrt{{\hat{\Sigma }}_{\tau }(x)/nh},\;{\hat{\mu }}_{\tau }(x)+z_{1-\alpha /2}\sqrt{{\hat{\Sigma }}_{\tau }(x)/nh}\right],$$

where $z_{1-\alpha /2}$ is the (1-α/2)-quantile of the standard normal distribution N(0,1).

### 3.2 | Bandwidth Selection

To select the optimal bandwidth h, we adopt the modified multi-fold cross-validation criterion proposed by Cai et al. (2000), also used in Xie et al. (2014) for varying-coefficient expectile model and Jiang et al. (2022) for single-index expectile model. The basic idea behind this method is that the modified cross-validation criterion is attentive to the structure of stationary time series data. Specifically, for two positive integers l and Q such that n>Ql, one can first use Q subseries of lengths n-ql for q=1,…,Q to estimate the unknown coefficient functions, and then compute the one-step forecast errors of other subseries of length l based on the estimated model. More precisely, the optimal bandwidth $h_{\text{opt}}$ is chosen by minimizing the average asymmetric mean squared error (AAMSE),

$$\text{AAMSE}(h)=\sum_{q=1}^{Q}{\text{AAMSE}}_{q}(h),$$

where ${\text{AAMSE}}_{q}(h)=l^{-1}\sum_{t=n-ql+1}^{n-ql+l}\Psi_{\tau }\left(Y_{t}-{\hat{\mu }}_{\tau }\left(X_{t}\right)\right)$ for q=1,…,Q, and ${\hat{\mu }}_{\tau }\left(X_{t}\right)$ is computed from the sample $\left\{\left(Y_{t},X_{t}\right)\;:\;1\leq t\leq \right.n-ql\}$ with bandwidth equal $h[n/(n-ql){]}^{-1/(2p+3)}$. Note that the bandwidth is rescaled for different sample size according to its optimal rate $h\propto n^{-1/(2p+3)}$ as suggested by Theorem 2. It worth mentioning that $\Psi_{\tau }(u)$ is a continuous function in u and $\overset{ˆ}{G}(y|x)$ is also continuous in h, the objective function AAMSE(h) in the above multi-fold cross-validation is continuous over h. Hence, we can adapt the bandwidth selection method proposed in Cai et al. (2000). As suggested in Cai et al. (2000), we can take l=[n/10] and Q=4 in practice.

The selection of bandwidth for the expectile estimator by IWLS method can be computed by using the same arguments, just replacing ${\hat{\mu }}_{\tau }\left(X_{t}\right)$ by ${\tilde{\mu }}_{\tau }\left(X_{t}\right)$ in ${\text{AAMSE}}_{q}(h)$. We omit the details to save space.

## 4 | Numerical Examples

### 4.1 | Simulations

We conduct Monte Carlo simulation studies to investigate the finite sample performance of the proposed estimator and compare with the existing IWLS estimator. The data are generated from two data generating processes (DGP):

**DGP 1:** $Y_{t}=0.6\;\text{sin}\left(X_{t}\right)+0.5e_{t}$,
**DGP 2:** $Y_{t}=0.6\;\text{sin}\left(X_{t}\right)+0.5\sqrt{1+X_{t}^{2}}e_{t}$,

for t=1,…,n+200 with the initial observation $Y_{0}=0$, where $X_{t}=Y_{t-1}$, and $e_{t}$ follows from two distributions: (i) $e_{t}\sim N(0,1)$ and (ii) $e_{t}\sim t_{5}$. The first 200 observations of each sample are discarded for eliminating the start-up effects.

The true conditional expectiles of $Y_{t}$ given $X_{t}=x_{0}$ is $\mu_{\tau }\left(x_{0}\right)=0.6\;\text{sin}\left(x_{0}\right)+\sigma \left(x_{0}\right)\mu_{\tau }\left(e_{t}\right)$, where $\sigma \left(x_{0}\right)=0.5$ and $\sigma \left(x_{0}\right)=0.5\sqrt{1+x_{0}^{2}}$ for DGP 1 and DGP 2, respectively. The expectile $\mu_{\tau }\left(e_{t}\right)$ of $e_{t}$ is obtained by solving the following equation (Jones 1994): $\frac{P_{e}(u)-uF_{e}(u)}{2\left[P(u)-uF_{e}(u)\right]+\left[u-P_{e}(+\infty )\right]}=\tau$, where $f_{e}(\cdot )$ and $F_{e}(\cdot )$ are the density and distribution functions of $e_{t}$, respectively, $P_{e}(u)=\int_{-\infty }^{u}vf_{e}(v)dv$, and $P_{e}(\infty )$ is the expectation of $e_{t}$. Please refer to Daouia et al. (2024) for more details on the computation of expectiles.

In this paper, we only experiment the local linear estimation (p=1). The used kernel is the Epanechnikov kernel $K(u)=0.75(1-\left.u^{2}\right)I(|u|\leq 1)$. For comparison, we estimate the conditional expectile function by the existing IWLS estimator ${\tilde{\mu }}_{\tau }\left(x_{0}\right)$, also with the local linear estimation. The optimal bandwidths for both the proposed estimator ${\hat{\mu }}_{\tau }\left(x_{0}\right)$ and the IWLS estimator ${\tilde{\mu }}_{\tau }\left(x_{0}\right)$ are selected by the modified multifold cross-validation criterion in Section 3.2.

We conduct NS = 1000 Monte Carlo replications for samples of sizes n=200,400 along with expectiles τ=0.10,0.25,0.50,0.75,0.90 and $x_{0}=-0.5,0,0.5$. We calculate the empirical bias and standard deviation of the estimator ${\hat{\mu }}_{\tau }\left(x_{0}\right)$ by

$$\text{Bias}={\text{NS}}^{-1}\sum_{j=1}^{\text{NS}}{\hat{\mu }}_{\tau }^{\left(j\right)}\left(x_{0}\right)-\mu_{\tau }\left(x_{0}\right),$$

$$\text{SD}=\sqrt{{\text{NS}}^{-1}\sum_{j=1}^{\text{NS}}{\left[{\hat{\mu }}_{\tau }^{\left(j\right)}\left(x_{0}\right)-{\bar{\overset{ˆ}{\mu }}}_{\tau }\left(x_{0}\right)\right]}^{2},}$$

where ${\bar{\overset{ˆ}{\mu }}}_{\tau }\left(x_{0}\right)={\text{NS}}^{-1}\sum_{j=1}^{\text{NS}}{\hat{\mu }}_{\tau }^{\left(j\right)}\left(x_{0}\right)$ is the mean of ${\hat{\mu }}_{\tau }^{\left(1\right)}\left(x_{0}\right),\ldots ,{\hat{\mu }}_{\tau }^{\left(\text{NS}\right)}\left(x_{0}\right)$. The average estimated standard error (“ESE”) is given by $\text{ESE}={\text{NS}}^{-1}\sum_{j=1}^{\text{NS}}\sqrt{{\hat{\Sigma }}_{\tau }^{\left(j\right)}(x)/nh}$, where ${\hat{\Sigma }}_{\tau }^{\left(j\right)}(x)$ is given by (4). The 95% empirical coverage probability (“CP”) and the mean length of 95% confidence interval (“CI.len”) of ${\hat{\mu }}_{\tau }\left(Y_{t}|x_{0}\right)$ are computed by its (1-α) Wald confidence interval in (5) by taking α=5%. We also compute the mean squared errors (“MSE”) by $\text{MSE}={\text{NS}}^{-1}\sum_{j=1}^{\text{NS}}{\left[{\hat{\mu }}_{\tau }^{\left(j\right)}\left(x_{0}\right)-\mu_{\tau }\left(x_{0}\right)\right]}^{2}$.

Tables 1–4 summarize estimating results, including Bias, SD, ESE, CP, MSE and CI.len for different DGPs with different errors. Several observations can follow from these tables. First, both our proposed estimator (“Proposed”) and the IWLS estimator (“IWLS”) have small biases for all scenarios. Besides, the average estimated standard errors are close to the empirical standard deviations, and the finite sample coverage probabilities are close to the nominal level 95%, which validates our asymptotic distributions. Second, our proposed estimator is more efficient than the IWLS estimator, because our proposed estimator has smaller MSEs than the IWLS estimator for all cases considered. Finally, our proposed estimator is more accurate than the IWLS estimator with shorter length of 95% confidence intervals. As expected, as sample size n increases from 200 to 400, the biases, standard deviations, MSE and mean length of 95% confidence interval tend to be smaller, and the empirical coverage probabilities tend to be closer to the nominal level 95%. Overall, the performance of our proposed estimator is better than the IWLS estimator across different model specifications and different expectile levels.

**FIGURE 1 |:**
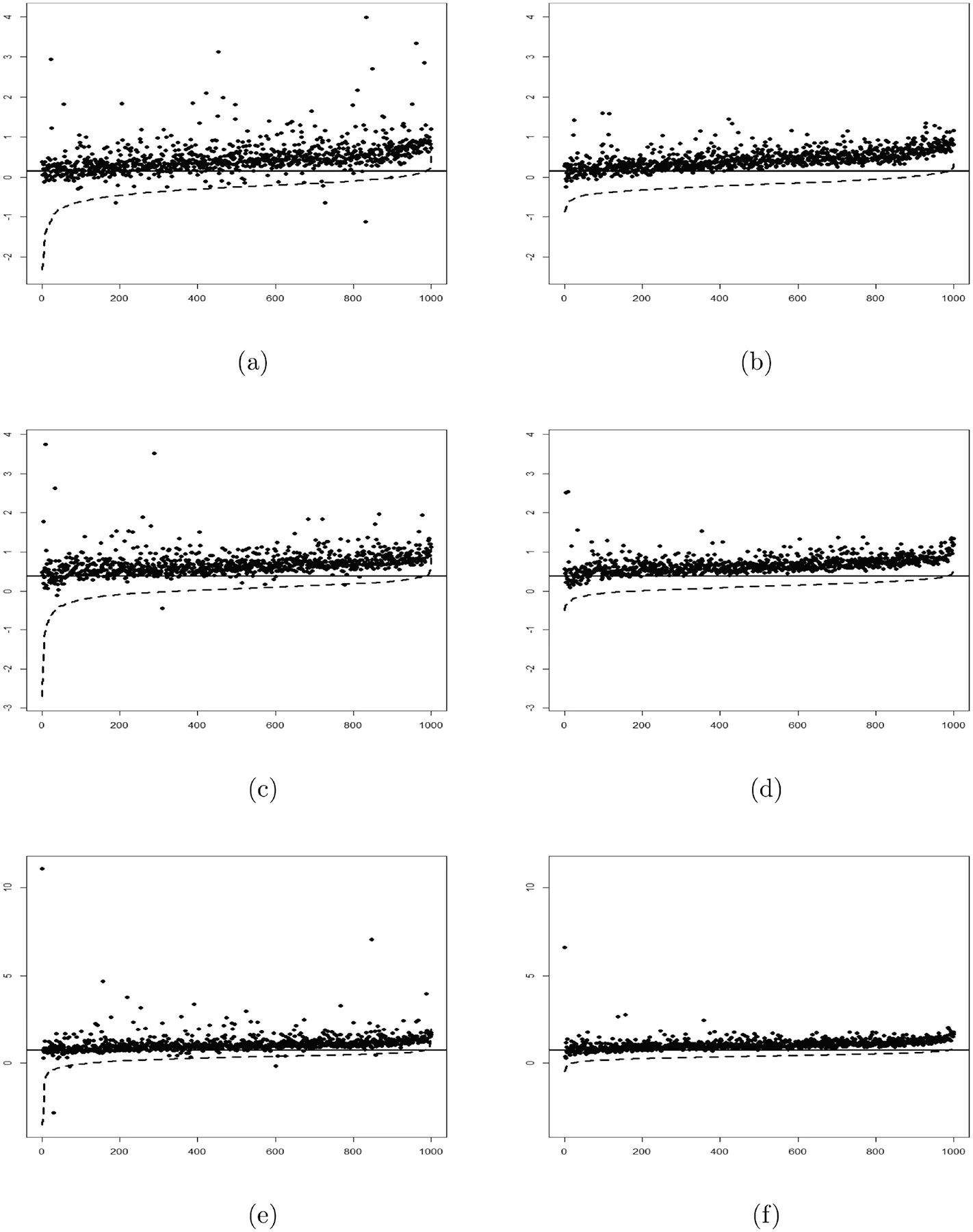
1000 realizations of IWLS CIs (left column) and proposed CIs (right column) when sample size n=200 for τ=0.75 expectile at different $x_{0}$ for DGP 2: $Y_{t}=0.6\;\text{sin}\left(X_{t}\right)+0.5\sqrt{1+X_{t}^{2}}e_{t}$ with $e_{t}\sim t_{5}$. In each figure, the horizontal solid line is the true conditional expectile, CIs are plotted by sorting in ascending order of the lower bounds (dashed lines) and the corresponding upper bounds (filled circles). The coverage probabilities in Table 4 are 0.936, 0.942, 0.923 and 0.919, 0928, 0.916 at $x_{0}=-0.5,0,0.5$ for the proposed and IWLS CIs, respectively. (a) IWLS CIs when $x_{0}=-0.5$. (b) Proposed CIs when $x_{0}=-0.5$. (c) IWLS CIs when $x_{0}=0.0$. (d) Proposed CIs when $x_{0}=0.0$. (e) IWLS CIs when $x_{0}=0.5$. (f) Proposed CIs when $x_{0}=0.5$.

To get a more insightful comparison between our proposed estimator and the IWLS estimator for a particular case when sample size n=200, we mimic fig. 2 in Xu (2013), which is also used in Fan and Liu (2016) for conditional quantile. Figure 1 plots the sequences proposed CIs and IWLS CIs (sorted according to the ascending order of the lower bounds) for τ=0.75 expectile at $x_{0}=-0.5,0,0.5$ for DGP 2: $Y_{t}=0.6\;\text{sin}\left(X_{t}\right)+0.5\sqrt{1+X_{t}^{2}}e_{t}$ with $e_{t}\sim t_{5}$, based on 1000 realizations. From Figure 1, one can clearly see the cases of rejections when the true conditional expectile is smaller than the lower bounds of CIs and is larger than the upper bounds of CIs. From Table 4, the coverage probabilities are actually 0.936, 0.942, 0.923 and 0.919, 0928, 0.916 at $x_{0}=-0.5,0,0.5$ for the proposed and IWLS CIs, respectively. It can be seen that IWLS CIs tend to have some extreme large upper and small lower bounds, and exhibit a biased center around the true conditional expectile. However, our proposed CIs are very stable in length and nicely centered around the true conditional expectile.

### 4.2 | An Empirical Application

**FIGURE 2 |:**
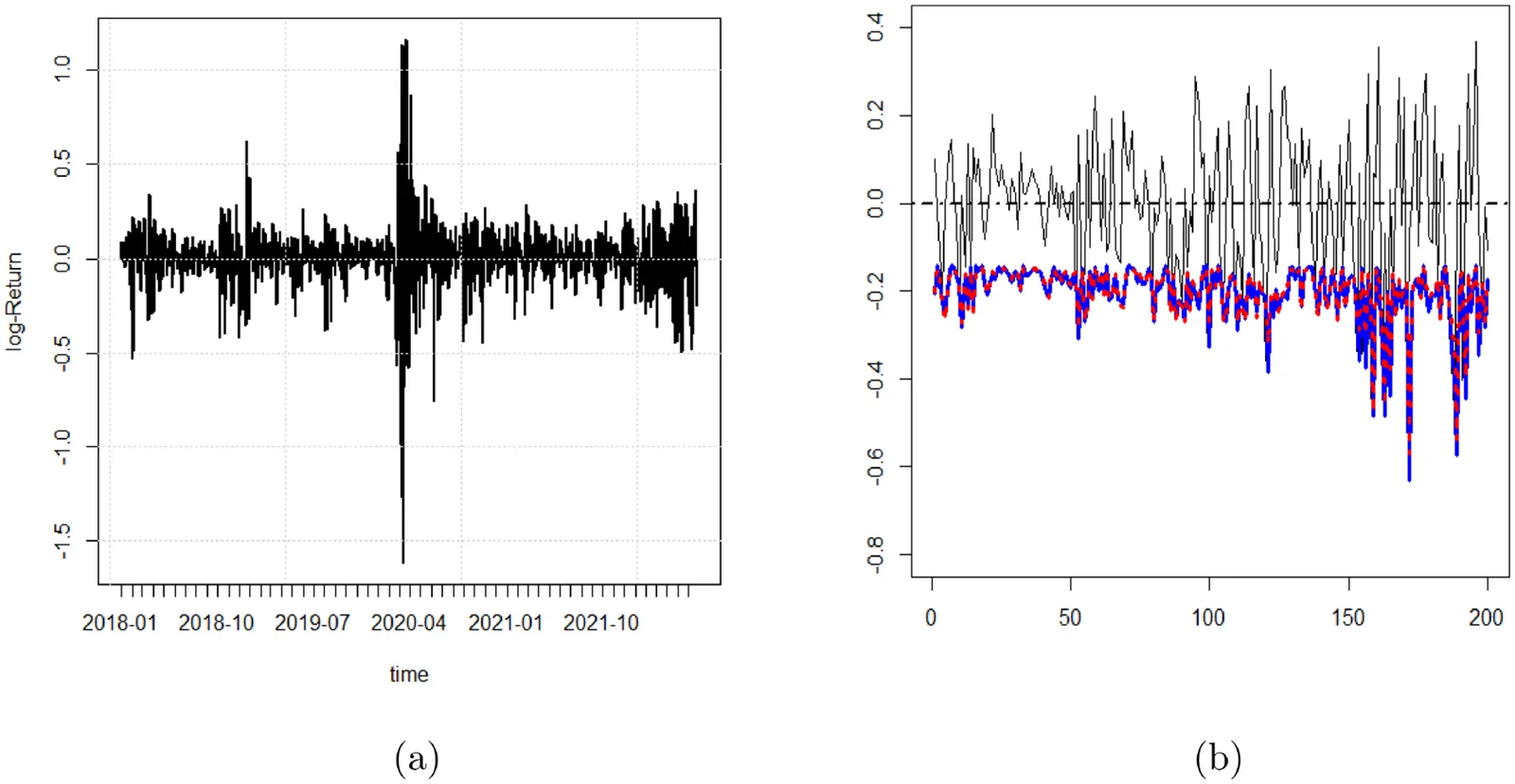
Analysis of S&P500 index data. (a) Time series plot of the daily return for S&P500 index data from January 2, 1980 to November 30, 2022. (b) The estimated 5% EVaR using our proposed method (*in blue*) and IWLS method (*in red*) for the last 500 out-of-sample observations of S&P500 index data. (a) Daily returns. (b) Estimated 5% EVaR.

In this section, we provide an illustrative empirical example to compare the performances of our proposed estimator with those of the IWLS estimator. The daily data of S&P500 index from January 2, 1980 to November 30, 2022 are obtained from https://investing.com. It covers the COVID-19 pandemic, with 10,825 observations. The daily returns are calculated by $Y_{t}=100\times \text{log}\left(P_{t}/P_{t-1}\right)$, where $P_{t}$ is the daily closing price of S&P500 index. Table 5 reports the descriptive statistics of S&P500 index returns. The sample mean is close to zero with slightly negative skewness. The augmented Dickey-Fuller (ADF) non-stationary test provides strong evidence for stationarity of $Y_{t}$. The large Jarque-Bera statistic rejects the null hypothesis that the joint distribution of $Y_{t}$ is normal. The time series plot of $Y_{t}$ in Figure 2a depicts that a major spike occurs at the beginning of 2020, when the COVID-19 pandemic broke out.

In this paper, we use the first-order lagged log return as the covariate, that is, $X_{t}=Y_{t-1}$. The goal is to estimate the τth expectile of the distribution of $Y_{t}$, which corresponds to the τ-level EVaR, conditional on a value of $X_{t}$. The first 10,324 observations from January 02, 1980 to December 06, 2020 are used as training data for model estimation, and the remaining $n_{1}=500$ observations are used for the out-of-sample evaluation. For comparison, we use the proposed estimator and the IWLS estimator. The implementation methods described in Section 3 are applied here, with the bandwidths selected by the modified multifold cross-validation for both the proposed estimator and the IWLS estimator.

As suggested in Bellini and Di Bernardino (2017), we consider the associated realized loss for the out-of-sample prediction,

$${ℒ}^{\text{EVaR}}(\tau )=n_{1}^{-1}\sum_{t=1}^{n_{1}}\Psi_{\tau }\left(Y_{t}-{\hat{\mu }}_{\tau }\left(X_{t}\right)\right),$$

where the τth conditional expectile ${\overset{ˆ}{\mu }}_{\tau }\left(X_{t}\right)$ of $Y_{t}$ evaluates at $X_{t}$ with ${\hat{\mu }}_{\tau }\left(X_{t}\right)$ estimated from in-sample data with 10,324 observations. Table 6 reports the associated realized loss ${ℒ}^{\text{EVaR}}(\tau )$ for τ=0.05,0.10,0.5,0.90,0.95. As expected, our proposed estimator is better than IWLS estimator for all expectile levels, because the associated realized losses of our proposed estimator is smaller than those of the IWLS estimator. Further, we also compare the computational time of the proposed estimator and IWLS estimator. As expected, the computational time of our proposed estimator is much less than that of IWLS estimator. For example, the computational time of our proposed estimator for ${ℒ}^{\text{EVaR}}(\tau )$ at τ=0.1 is 4496.26 s, which is about one-seventeen of that of IWLS method (80367.99 s). This is not surprise, because the IWLS estimator requires the iteration algorithm.

Finally, Figure 2b displays the estimated 5% EVaR for the last 500 out-of-sample observations of S&P500 index data, by using our proposed estimator and IWLS estimator. The estimated 5% EVaRs by two estimators exhibit a similar pattern.

## 5 | Extensions

Although this paper focuses on the case where covariate $X_{t}$ is univariate, the proposed method and its theory established above can be easily extended to a multivariate continuous $X_{t}$, with more complicated notations being involved. However, it is more challenging for the multivariate mixed continuous and discrete covariates $X_{t}$. To this end, we adopt the framework in Racine and Li (2004) and Li and Racine (2008). We consider a q-dimensional vector of covariates $X_{t}={\left(X_{1t}^{c},\ldots ,X_{q_{1}t}^{c},X_{1t}^{d},\ldots ,X_{q_{2}t}^{d}\right)}^{⊤}$ with the first $q_{1}$ variables being continuous and the last $q_{2}$ variables being discrete, where $q=q_{1}+q_{2}$ and $x={\left(x_{1}^{c},\ldots ,x_{q_{1}}^{c},x_{1}^{d},\ldots ,x_{q_{2}}^{d}\right)}^{⊤}$.

Following Racine and Li (2004), we introduce a generalized product kernel for mixed data,

$${𝒦}_{h,\lambda }\left(X_{t},x\right)=\prod_{j=1}^{q_{1}}K_{h_{j}}\left(X_{jt}^{c}-x_{j}^{c}\right)\prod_{l=1}^{q_{2}}L_{\lambda_{l}}\left(X_{lt}^{d},x_{l}^{d}\right),$$

where $L_{\lambda_{l}}\left(X_{lt}^{d},x_{l}^{d}\right)=I\left(X_{lt}^{d}=x_{l}^{d}\right)+\lambda_{l}I\left(X_{lt}^{d}\neq x_{l}^{d}\right)$ and $\lambda_{l}$’s are smoothing bandwidths for discrete covariates. Similar to (2), we can define the local polynomial estimator of G(y∣x) as

(6) $$\hat{G}(y|x)=\frac{{\hat{G}}_{1}(y|x)}{{\hat{G}}_{2}(y|x)}≔\frac{n^{-1}\sum_{t=1}^{n}{𝒲}_{h,\lambda }\left(X_{t}-x\right){\left(Y_{t}-y\right)}_{-}}{n^{-1}\sum_{t=1}^{n}{𝒲}_{h,\lambda }\left(X_{t}-x\right){\left(Y_{t}-y\right)}_{+}},$$

where ${𝒲}_{h,\lambda }\left(X_{t}-x\right)=e_{1}^{⊤}H^{-1}S_{n}^{-1}H^{-1}{\left(1,X_{t}-x,\ldots ,{\left(X_{t}-x\right)}^{p}\right)}^{⊤}{𝒦}_{h,\lambda }\left(X_{t},x\right)$. Consequently, one can estimate the conditional expectile $\mu_{\tau }(Y|x)$ by

$${\hat{\mu }}_{\tau }(x)=\text{inf}\left\{y\in R\;:\;\hat{G}(y|x)\geq \frac{\tau }{1-\tau }\right\},$$

Note that for IID data, Racine and Li (2004) and Li and Racine (2008) established the asymptotic theory for the local constant estimator of nonparametric regression and F(y∣x), respectively. We can generalize their results to time series data for the proposed estimator. However, it requires additional efforts to handle discrete covariates, which is beyond the scope of this paper, and further investigation is needed for future study.

The proposed method can be also generalized to a semi-parametric model. Without loss of generality, we consider the following partially linear expectile regression model,

(7) $$\mu_{\tau }\left(X_{t}\right)=X_{1t}^{⊤}\beta_{\tau }+g\left(X_{2t}\right),$$

where $X_{t}={\left(X_{1t},X_{2t}\right)}^{⊤}$ with $X_{1t}$ being a d-dimensional covariates and $X_{2t}$ being a univariate covariate, $\beta_{\tau }$ is an unknown d-dimensional regression parameters, and g(⋅) is an unknown smooth function. Adam and Gijbels (2021) proposed a local polynomial fitting for model (7). One possible algorithm using our proposed method is to estimate $\beta_{\tau }$ and g(⋅) iteratively. First, given ${\hat{\beta }}_{\tau }^{\left(k\right)}$ and $X_{t2}=x_{2}$, we estimate $g\left(x_{2}\right)$ by ${\hat{g}}^{\left(k\right)}\left(x_{2}\right)=\text{inf}\{y\in R\;:\;\left.\hat{G}\left(y|x_{2},{\hat{\beta }}_{\tau }^{\left(k\right)}\right)\geq \tau /(1-\tau )\right\}$, where

$$\hat{G}\left(y|x_{2},\beta \right)=\frac{{\hat{G}}_{1}\left(y|x_{2},\beta \right)}{{\hat{G}}_{2}\left(y|x_{2},\beta \right)}≔\frac{n^{-1}\sum_{l=t}^{n}W_{p}\left\{\left(X_{2t}-x_{2}\right)/h\right\}{\left({\tilde{Y}}_{t}(\beta )-y\right)}_{-}}{n^{-1}\sum_{l=t}^{n}W_{p}\left\{\left(X_{2t}-x_{2}\right)/h\right\}{\left({\tilde{Y}}_{t}(\beta )-y\right)}_{+}},$$

and ${\tilde{Y}}_{t}(\beta )=Y_{t}-X_{1t}^{⊤}\beta$. Then, we update $\beta_{\tau }$ by

$${\overset{ˆ}{\beta }}_{\tau }^{\left(k+1\right)}={\left\{\sum_{t=1}^{n}\omega_{\tau }\left(t,{\hat{g}}^{\left(k\right)},{\hat{\beta }}_{\tau }^{\left(k\right)}\right)X_{1t}X_{1t}^{⊤}\right\}}^{-1}\times \left\{\sum_{t=1}^{n}\omega_{\tau }\left(t,{\hat{g}}^{\left(k\right)},{\hat{\beta }}_{\tau }^{\left(k\right)}\right)X_{1t}\left(Y_{t}-{\hat{g}}^{\left(k\right)}\left(X_{2t}\right)\right)\right\},$$

where $\omega_{\tau }(t,g,\beta )=\left|\tau -I\left\{Y_{t}-g\left(X_{2t}\right)\leq X_{1t}^{⊤}\beta \right\}\right|$. Then, the proposed estimation can be obtained by repeating the above estimation until convergence. We leave this extension for future study.

## 6 | Concluding Remarks

In this article, we propose a new and direct nonparametric method for estimating conditional expectile functions with large-scale time series data. Our proposed estimator is obtained by inverting a local polynomial estimator of the conditional loss-gain ratio function, which is easy-to-implement. The proposed conditional expectile estimator is shown to be both computationally and statistically more efficient than the existing expectile-loss-based conditional expectile estimators, which require IWLS algorithms. Our numerical studies confirm that the proposed Wald-type confidence interval of the proposed estimator has better coverage probabilities and narrower lengths than the existing estimator. An empirical application demonstrate the appealing feature of our method in good finite sample performance and computationally efficiency for large-scale time series data.

Given the simplicity and superior performance of the proposed method to the existing methods, it would be worthwhile investigating the extension of this method to more general settings. For example, one can allow for the case with multidimensional covariates, as in Li and Racine (2008), or allow for nonstationary time series considered in Zhou and Wu (2009). Another extension is to consider the multidimensional expectile in Maume-Deschamps et al. (2017). We leave these topics as possible future research study.

## Supplementary Material

Supplemental Material

Additional supporting information can be found online in the Supporting Information section. **Data S1**. Supporting Information.

## Figures and Tables

**TABLE 1 |:** Performance of the proposed estimator ${\hat{\mu }}_{\tau }\left(x_{0}\right)$ and IWLS estimator ${\tilde{\mu }}_{\tau }\left(x_{0}\right)$ at $x_{0}=-0.5,0.0,0.5$ based on 1000 simulated samples of n=200,400 observations for DGP 1: $Y_{t}=0.6\;\text{sin}\left(X_{t}\right)+0.5e_{t}$ with $e_{t}\sim N(0,1)$.

τ	$x_{0}$	n=200						n=400					
		Proposed			IWLS			Proposed			IWLS		
		−0.5	0.0	0.5	−0.5	0.0	0.5	−0.5	0.0	0.5	−0.5	0.0	0.5
0.10	True	−0.897	−0.609	−0.322	−0.897	−0.609	−0.322	−0.897	−0.609	−0.322	−0.897	−0.609	−0.322
	Bias	0.024	0.012	0.006	0.022	0.014	0.011	0.014	0.001	−0.001	0.013	0.005	0.004
	SD	0.125	0.108	0.116	0.136	0.118	0.129	0.090	0.076	0.081	0.095	0.082	0.085
	ESE	0.111	0.102	0.109	0.118	0.109	0.118	0.080	0.073	0.078	0.084	0.077	0.083
	CP	0.892	0.909	0.922	0.895	0.903	0.913	0.895	0.938	0.938	0.900	0.943	0.939
	MSE	0.016	0.012	0.013	0.019	0.014	0.017	0.008	0.006	0.007	0.009	0.007	0.007
	CI.len	0.436	0.400	0.428	0.462	0.427	0.462	0.314	0.287	0.305	0.330	0.303	0.325
0.25	True	−0.596	−0.309	−0.021	−0.596	−0.309	−0.021	−0.596	−0.309	−0.021	−0.596	−0.309	−0.021
	Bias	0.019	0.009	0.005	0.012	0.007	0.004	0.013	0.002	−0.002	0.011	0.002	−0.001
	SD	0.110	0.094	0.100	0.123	0.103	0.115	0.079	0.068	0.071	0.084	0.071	0.078
	ESE	0.099	0.090	0.097	0.105	0.096	0.105	0.070	0.064	0.069	0.074	0.068	0.073
	CP	0.910	0.939	0.939	0.910	0.932	0.929	0.908	0.940	0.940	0.917	0.945	0.934
	MSE	0.012	0.009	0.010	0.015	0.011	0.013	0.006	0.005	0.005	0.007	0.005	0.006
	CI.len	0.387	0.351	0.380	0.412	0.377	0.412	0.274	0.249	0.269	0.289	0.265	0.287
0.50	True	−0.288	0.000	0.288	−0.288	0.000	0.288	−0.288	0.000	0.288	−0.288	0.000	0.288
	Bias	0.019	0.012	0.005	0.008	0.003	−0.004	0.016	0.007	−0.001	0.009	0.001	−0.006
	SD	0.104	0.091	0.098	0.115	0.099	0.109	0.075	0.064	0.068	0.080	0.070	0.074
	ESE	0.094	0.086	0.094	0.100	0.092	0.100	0.066	0.061	0.066	0.070	0.065	0.070
	CP	0.920	0.933	0.944	0.915	0.935	0.937	0.917	0.946	0.947	0.928	0.945	0.945
	MSE	0.011	0.008	0.010	0.013	0.010	0.012	0.006	0.004	0.005	0.006	0.005	0.006
	CI.len	0.370	0.338	0.369	0.393	0.359	0.394	0.259	0.238	0.258	0.276	0.254	0.275
0.75	True	0.021	0.309	0.596	0.021	0.309	0.596	0.021	0.309	0.596	0.021	0.309	0.596
	Bias	0.018	0.015	0.010	0.002	−0.002	−0.008	0.017	0.011	0.002	0.006	0.000	−0.008
	SD	0.109	0.099	0.108	0.121	0.105	0.118	0.077	0.069	0.074	0.080	0.073	0.077
	ESE	0.099	0.092	0.101	0.106	0.097	0.107	0.070	0.065	0.070	0.073	0.068	0.073
	CP	0.923	0.925	0.933	0.909	0.924	0.930	0.924	0.929	0.934	0.922	0.930	0.940
	MSE	0.012	0.010	0.012	0.015	0.011	0.014	0.006	0.005	0.006	0.006	0.005	0.006
	CI.len	0.390	0.360	0.396	0.415	0.381	0.418	0.273	0.253	0.276	0.287	0.265	0.287
0.90	True	0.322	0.609	0.897	0.322	0.609	0.897	0.322	0.609	0.897	0.322	0.609	0.897
	Bias	0.018	0.018	0.016	−0.008	−0.009	−0.015	0.016	0.016	0.005	−0.000	−0.002	−0.013
	SD	0.130	0.118	0.130	0.137	0.124	0.131	0.088	0.081	0.086	0.092	0.085	0.088
	ESE	0.115	0.107	0.117	0.120	0.111	0.120	0.081	0.076	0.082	0.084	0.079	0.084
	CP	0.899	0.915	0.920	0.891	0.916	0.925	0.917	0.931	0.937	0.917	0.931	0.923
	MSE	0.017	0.014	0.017	0.019	0.015	0.017	0.008	0.007	0.007	0.008	0.007	0.008
	CI.len	0.449	0.420	0.459	0.468	0.435	0.472	0.317	0.297	0.321	0.329	0.308	0.329

Abbreviations: Bias: empirical bias; SD: empirical standard error; ESE: average estimated standard error; CP: 95% coverage probability; MSE: mean squared error; CI.len: average length of 95% confidence interval.

**TABLE 2 |:** Performance of the proposed estimator ${\hat{\mu }}_{\tau }\left(x_{0}\right)$ and IWLS estimator ${\tilde{\mu }}_{\tau }\left(x_{0}\right)$ at $x_{0}=-0.5,0.0,0.5$ based on 1000 simulated samples of n=200,400 observations for DGP 1: $Y_{t}=0.6\;\text{sin}\left(X_{t}\right)+0.5e_{t}$ with $e_{t}\sim t_{5}$.

τ	$x_{0}$	n=200						n=400					
		Proposed			IWLS			Proposed			IWLS		
		−0.5	0.0	0.5	−0.5	0.0	0.5	−0.5	0.0	0.5	−0.5	0.0	0.5
0.10	True	−1.049	−0.761	−0.474	−1.049	−0.761	−0.474	−1.049	−0.761	−0.474	−1.049	−0.761	−0.474
	Bias	0.020	0.008	−0.001	0.009	0.010	0.002	0.009	−0.004	−0.002	0.004	0.002	0.001
	SD	0.198	0.172	0.199	0.227	0.192	0.224	0.135	0.131	0.134	0.157	0.146	0.160
	ESE	0.182	0.166	0.177	0.198	0.180	0.196	0.131	0.119	0.127	0.144	0.129	0.140
	CP	0.901	0.907	0.914	0.901	0.906	0.900	0.918	0.917	0.912	0.916	0.912	0.902
	MSE	0.040	0.030	0.040	0.052	0.037	0.050	0.018	0.017	0.018	0.025	0.021	0.026
	CI.len	0.713	0.652	0.695	0.775	0.706	0.769	0.513	0.466	0.500	0.563	0.507	0.550
0.25	True	−0.659	−0.372	−0.084	−0.659	−0.372	−0.084	−0.659	−0.372	−0.084	−0.659	−0.372	−0.084
	Bias	0.014	0.006	−0.002	0.005	0.005	−0.001	0.006	−0.003	−0.004	0.002	−0.005	−0.003
	SD	0.141	0.124	0.143	0.172	0.143	0.168	0.100	0.091	0.097	0.110	0.104	0.116
	ESE	0.135	0.123	0.132	0.150	0.136	0.150	0.096	0.087	0.094	0.106	0.096	0.104
	CP	0.928	0.942	0.938	0.926	0.938	0.936	0.930	0.939	0.936	0.933	0.944	0.931
	MSE	0.020	0.015	0.020	0.029	0.021	0.028	0.010	0.008	0.009	0.012	0.011	0.013
	CI.len	0.530	0.481	0.519	0.589	0.533	0.587	0.378	0.342	0.369	0.417	0.378	0.408
0.50	True	−0.288	0.000	0.288	−0.288	0.000	0.288	−0.288	0.000	0.288	−0.288	0.000	0.288
	Bias	0.011	0.008	0.002	0.002	−0.002	−0.004	0.008	−0.001	−0.002	0.001	−0.007	−0.005
	SD	0.129	0.112	0.133	0.160	0.131	0.154	0.091	0.079	0.086	0.101	0.092	0.103
	ESE	0.121	0.111	0.121	0.135	0.124	0.135	0.086	0.078	0.085	0.095	0.087	0.095
	CP	0.933	0.956	0.926	0.929	0.951	0.930	0.932	0.947	0.950	0.936	0.947	0.939
	MSE	0.017	0.013	0.018	0.025	0.017	0.024	0.008	0.006	0.007	0.010	0.009	0.011
	CI.len	0.474	0.435	0.476	0.527	0.484	0.528	0.335	0.307	0.335	0.373	0.341	0.373
0.75	True	0.084	0.372	0.659	0.084	0.372	0.659	0.084	0.372	0.659	0.084	0.372	0.659
	Bias	0.011	0.012	0.007	−0.011	−0.004	−0.011	0.009	0.002	0.001	−0.002	−0.010	−0.010
	SD	0.145	0.132	0.161	0.170	0.170	0.187	0.106	0.087	0.099	0.119	0.101	0.117
	ESE	0.133	0.125	0.139	0.146	0.138	0.151	0.096	0.088	0.098	0.105	0.096	0.107
	CP	0.936	0.942	0.923	0.919	0.928	0.916	0.931	0.944	0.942	0.923	0.936	0.926
	MSE	0.021	0.018	0.026	0.029	0.029	0.035	0.011	0.008	0.010	0.014	0.010	0.014
	CI.len	0.522	0.489	0.544	0.571	0.543	0.591	0.375	0.346	0.383	0.410	0.378	0.418
0.90	True	0.474	0.761	1.049	0.474	0.761	1.049	0.474	0.761	1.049	0.474	0.761	1.049
	Bias	0.006	0.013	0.043	−0.026	−0.014	−0.016	0.010	0.004	0.008	−0.007	−0.012	−0.014
	SD	0.206	0.193	0.725	0.220	0.241	0.284	0.157	0.128	0.148	0.169	0.139	0.159
	ESE	0.181	0.172	0.195	0.188	0.181	0.200	0.132	0.122	0.138	0.140	0.129	0.144
	CP	0.907	0.913	0.889	0.884	0.891	0.862	0.913	0.922	0.927	0.897	0.902	0.910
	MSE	0.042	0.037	0.527	0.049	0.058	0.081	0.025	0.016	0.022	0.028	0.019	0.026
	CI.len	0.708	0.673	0.763	0.738	0.710	0.783	0.518	0.479	0.542	0.548	0.507	0.563

Abbreviations: Bias: empirical bias; SD: empirical standard error; ESE: average estimated standard error; CP: 95% coverage probability; MSE: mean squared error; CI.len: average length of 95% confidence interval.

**TABLE 3 |:** Performance of the proposed estimator ${\hat{\mu }}_{\tau }\left(x_{0}\right)$ and IWLS estimator ${\tilde{\mu }}_{\tau }\left(x_{0}\right)$ at $x_{0}=-0.5,0.0,0.5$ based on 1000 simulated samples of n=200,400 observations for DGP 2: $Y_{t}=0.6\;\text{sin}\left(X_{t}\right)+0.5\sqrt{1+X_{t}^{2}}e_{t}$ with $e_{t}\sim N(0,1)$.

τ	$x_{0}$	n=200						n=400					
		Proposed			IWLS			Proposed			IWLS		
		−0.5	0.0	0.5	−0.5	0.0	0.5	−0.5	0.0	0.5	−0.5	0.0	0.5
0.10	True	−0.969	−0.609	−0.393	−0.969	−0.609	−0.393	−0.969	−0.609	−0.393	−0.969	−0.609	−0.393
	Bias	0.009	0.002	0.006	0.020	0.010	0.008	−0.000	−0.011	−0.003	0.007	−0.003	0.003
	SD	0.151	0.115	0.139	0.181	0.140	0.173	0.105	0.084	0.094	0.119	0.098	0.112
	ESE	0.138	0.113	0.129	0.156	0.128	0.151	0.099	0.081	0.093	0.110	0.091	0.106
	CP	0.908	0.933	0.916	0.912	0.922	0.904	0.921	0.924	0.932	0.926	0.931	0.923
	MSE	0.023	0.013	0.019	0.033	0.020	0.030	0.011	0.007	0.009	0.014	0.010	0.012
	CI.len	0.540	0.442	0.506	0.612	0.503	0.593	0.388	0.318	0.365	0.431	0.358	0.416
0.25	True	−0.633	−0.309	−0.057	−0.633	−0.309	−0.057	−0.633	−0.309	−0.057	−0.633	−0.309	−0.057
	Bias	0.012	0.007	0.004	0.011	0.004	0.002	0.008	−0.002	−0.003	0.006	−0.002	−0.002
	SD	0.130	0.100	0.121	0.166	0.127	0.158	0.091	0.072	0.081	0.110	0.084	0.099
	ESE	0.120	0.099	0.115	0.142	0.117	0.139	0.085	0.070	0.082	0.097	0.081	0.096
	CP	0.925	0.940	0.927	0.916	0.934	0.931	0.921	0.947	0.937	0.918	0.948	0.939
	MSE	0.017	0.010	0.015	0.028	0.016	0.025	0.008	0.005	0.007	0.012	0.007	0.010
	CI.len	0.472	0.388	0.452	0.557	0.458	0.543	0.332	0.275	0.320	0.380	0.318	0.375
0.50	True	−0.288	0.000	0.288	−0.288	0.000	0.288	−0.288	0.000	0.288	−0.288	0.000	0.288
	Bias	0.015	0.014	0.009	0.001	−0.001	−0.004	0.014	0.007	0.002	0.006	−0.001	−0.003
	SD	0.126	0.100	0.120	0.164	0.127	0.152	0.087	0.068	0.080	0.105	0.083	0.100
	ESE	0.115	0.096	0.114	0.137	0.113	0.135	0.080	0.068	0.080	0.093	0.078	0.093
	CP	0.919	0.939	0.939	0.919	0.945	0.938	0.913	0.947	0.946	0.922	0.948	0.943
	MSE	0.016	0.010	0.015	0.027	0.016	0.023	0.008	0.005	0.006	0.011	0.007	0.010
	CI.len	0.449	0.378	0.447	0.535	0.443	0.530	0.313	0.265	0.313	0.364	0.306	0.364
0.75	True	0.057	0.309	0.633	0.057	0.309	0.633	0.057	0.309	0.633	0.057	0.309	0.633
	Bias	0.018	0.023	0.019	−0.003	−0.003	−0.009	0.018	0.017	0.012	0.006	0.003	−0.003
	SD	0.132	0.111	0.137	0.163	0.133	0.162	0.094	0.075	0.089	0.108	0.084	0.105
	ESE	0.122	0.104	0.125	0.141	0.117	0.141	0.085	0.073	0.087	0.096	0.081	0.098
	CP	0.925	0.934	0.930	0.920	0.936	0.920	0.923	0.940	0.939	0.917	0.944	0.936
	MSE	0.018	0.013	0.019	0.027	0.018	0.026	0.009	0.006	0.008	0.012	0.007	0.011
	CI.len	0.478	0.406	0.490	0.552	0.457	0.551	0.332	0.285	0.343	0.376	0.319	0.382
0.90	True	0.393	0.609	0.969	0.393	0.609	0.969	0.393	0.609	0.969	0.393	0.609	0.969
	Bias	0.008	0.029	0.028	−0.013	−0.008	−0.017	0.018	0.027	0.022	0.005	0.005	−0.005
	SD	0.170	0.143	0.169	0.179	0.155	0.174	0.116	0.092	0.109	0.124	0.099	0.117
	ESE	0.145	0.123	0.151	0.153	0.128	0.153	0.101	0.087	0.106	0.107	0.092	0.110
	CP	0.894	0.909	0.913	0.895	0.913	0.901	0.910	0.928	0.935	0.924	0.942	0.930
	MSE	0.029	0.021	0.029	0.032	0.024	0.031	0.014	0.009	0.012	0.016	0.010	0.014
	CI.len	0.568	0.484	0.593	0.601	0.502	0.601	0.397	0.341	0.416	0.420	0.360	0.431

Abbreviations: Bias: empirical bias; SD: empirical standard error; ESE: average estimated standard error; CP: 95% coverage probability; MSE: mean squared error; CI.len: average length of 95% confidence interval.

**TABLE 4 |:** Performance of the proposed estimator ${\hat{\mu }}_{\tau }\left(x_{0}\right)$ and IWLS estimator ${\tilde{\mu }}_{\tau }\left(x_{0}\right)$ at $x_{0}=-0.5,0.0,0.5$ based on 1000 simulated samples of n=200,400 observations for DGP 2: $Y_{t}=0.6\text{sin}\left(X_{t}\right)+0.5\sqrt{1+X_{t}^{2}}e_{t}$ with $e_{t}\sim t_{5}$.

τ	$x_{0}$	n=200						n=400					
		Proposed			IWLS			Proposed			IWLS		
		−0.5	0.0	0.5	−0.5	0.0	0.5	−0.5	0.0	0.5	−0.5	0.0	0.5
0.10	True	−1.139	−0.761	−0.564	−1.139	−0.761	−0.564	−1.139	−0.761	−0.564	−1.139	−0.761	−0.564
	Bias	0.005	0.004	0.002	0.007	0.017	0.023	−0.012	−0.018	−0.004	0.004	−0.010	0.015
	SD	0.248	0.193	0.247	0.328	0.246	0.345	0.175	0.142	0.171	0.226	0.192	0.222
	ESE	0.225	0.184	0.215	0.260	0.214	0.255	0.165	0.135	0.156	0.195	0.163	0.189
	CP	0.892	0.923	0.878	0.874	0.890	0.864	0.926	0.935	0.893	0.909	0.914	0.870
	MSE	0.061	0.037	0.061	0.107	0.061	0.120	0.031	0.021	0.029	0.051	0.037	0.050
	CI.len	0.880	0.722	0.843	1.020	0.840	1.002	0.646	0.528	0.613	0.763	0.640	0.740
0.25	True	−0.703	−0.372	−0.128	−0.703	−0.372	−0.128	−0.703	−0.372	−0.128	−0.703	−0.372	−0.128
	Bias	0.006	0.007	0.001	−0.002	0.005	0.013	−0.003	−0.010	−0.004	0.000	−0.011	0.008
	SD	0.181	0.141	0.177	0.269	0.196	0.305	0.127	0.103	0.124	0.171	0.144	0.165
	ESE	0.168	0.140	0.163	0.209	0.173	0.207	0.120	0.099	0.115	0.151	0.127	0.146
	CP	0.920	0.945	0.929	0.911	0.932	0.913	0.938	0.945	0.929	0.927	0.932	0.916
	MSE	0.033	0.020	0.031	0.072	0.038	0.093	0.016	0.011	0.015	0.029	0.021	0.027
	CI.len	0.659	0.547	0.638	0.817	0.677	0.812	0.472	0.389	0.452	0.593	0.496	0.571
0.50	True	−0.288	0.000	0.288	−0.288	0.000	0.288	−0.288	0.000	0.288	−0.288	0.000	0.288
	Bias	0.011	0.016	0.008	−0.015	−0.005	0.001	0.005	0.002	0.000	−0.002	−0.006	−0.002
	SD	0.164	0.129	0.164	0.251	0.195	0.294	0.113	0.091	0.110	0.159	0.128	0.152
	ESE	0.150	0.127	0.152	0.188	0.161	0.196	0.106	0.090	0.106	0.137	0.115	0.136
	CP	0.928	0.945	0.935	0.912	0.942	0.916	0.938	0.942	0.942	0.937	0.935	0.935
	MSE	0.027	0.017	0.027	0.063	0.038	0.086	0.013	0.008	0.012	0.025	0.016	0.023
	CI.len	0.587	0.500	0.594	0.738	0.629	0.767	0.415	0.351	0.415	0.536	0.452	0.535
0.75	True	0.128	0.372	0.703	0.128	0.372	0.703	0.128	0.372	0.703	0.128	0.372	0.703
	Bias	0.010	0.027	0.023	−0.031	−0.007	−0.011	0.006	0.013	0.012	−0.009	−0.004	−0.008
	SD	0.183	0.150	0.202	0.278	0.211	0.337	0.128	0.104	0.129	0.171	0.139	0.161
	ESE	0.165	0.145	0.176	0.199	0.172	0.214	0.118	0.102	0.123	0.146	0.124	0.149
	CP	0.925	0.948	0.935	0.883	0.930	0.906	0.935	0.946	0.942	0.930	0.924	0.925
	MSE	0.033	0.023	0.041	0.078	0.045	0.114	0.017	0.011	0.017	0.029	0.019	0.026
	CI.len	0.646	0.568	0.691	0.779	0.676	0.839	0.464	0.402	0.483	0.571	0.485	0.582
0.90	True	0.564	0.761	1.139	0.564	0.761	1.139	0.564	0.761	1.139	0.564	0.761	1.139
	Bias	0.004	0.039	0.056	−0.051	−0.010	−0.023	0.005	0.024	0.021	−0.020	−0.002	−0.019
	SD	0.271	0.233	0.511	0.333	0.277	0.429	0.189	0.152	0.188	0.226	0.187	0.221
	ESE	0.229	0.202	0.251	0.241	0.219	0.263	0.165	0.145	0.176	0.186	0.162	0.190
	CP	0.878	0.918	0.889	0.847	0.889	0.864	0.902	0.932	0.913	0.894	0.903	0.877
	MSE	0.073	0.056	0.264	0.113	0.077	0.185	0.036	0.024	0.036	0.051	0.035	0.049
	CI.len	0.897	0.790	0.984	0.946	0.859	1.030	0.648	0.569	0.691	0.731	0.635	0.743

Abbreviations: Bias: empirical bias; SD: empirical standard error; ESE: average estimated standard error; CP: 95% coverage probability; MSE: mean squared error; CI.len: average length of 95% confidence interval.

**TABLE 5 |:** Descriptive statistics of log returns for S&P500 index data.

Mean	Median	Min	Max	SD	Skewness	Kurtosis	ADF	JB
0.0004	0.0006	−2.048	1.158	1.136	−0.706	2.219	−2.290**	16719.361***

Abbreviations: ADF: the augmented Dickey-Fuller test statistic; JB: Jarque-Bera test statistic.

***, ** denote being significant at the 1%, 5% level.

**TABLE 6 |:** Realized loss function ${ℒ}^{\text{EVaR}}(\tau )$ and computational time (in seconds) for S&P500 index data using our proposed method and IWLS method.

τ	Proposed		IWLS	
	${ℒ}^{\text{EVaR}}(\tau )$	Time	${ℒ}^{\text{EVaR}}(\tau )$	Time
0.05	0.291	4501.38	0.295	90906.70
0.10	0.428	4496.26	0.442	80367.99
0.50	0.736	4510.09	0.834	28971.48
0.90	0.410	4503.06	0.477	80828.22
0.95	0.279	4484.36	0.351	90126.42

## Data Availability

The data that support the findings of the emiprical study are publically available at https://cn.investing.com.

## Footnotes

1 If $Y_{t}$ is continuous and the conditional density function of $Y_{t}$ given x exists, then G(y∣x) is continuous in y and strictly increasing. In this case, $\mu_{\tau }(x)=G^{-1}\left(\left.\frac{\tau }{1-\tau }\right|x\right)$, where $G^{-1}(\cdot |x)$ is the inverse function of G(⋅∣x). In practice, G(⋅∣x) is unknown and needed to be estimated using Equation (2). An efficient algorithm is also provided in Section 2.2.
