# Supplementary material for: Nonparametric Inference of Conditional Expectile Functions in Large-Scale Time Series Data With Improved Efficiency
Source: J Time Ser Anal. Author manuscript; Available in PMC 2026 Sep 12. (PMC13564231; doi:10.1111/jtsa.70021)
Supplement: Supplementary file 1 [file NIHMS2204381-supplement-Supplemental_Material.pdf]

Supplemental Material for "**Nonparametric inference of conditional expectile functions in large-scale time series data with improved efficiency**"

Feipeng Zhang *[1] and Ping-Shou Zhong †[2]

[1]School of Economics and Finance, Xi'an Jiaotong University, Xi'an, 710049, China

[2]Department of Mathematics, Statistics, and Computer Science, University of Illinois Chicago, 851 S. Morgan St., Chicago, Illinois, 60607

Abstract

In this supplemental material, we include detailed technical proofs for all the theoretical results presented in the paper.

Appendix A Some Useful Lemmas

For proving Theorem 1, we provide a result for general regression form

$$m(y|x) = \mathrm{E}\left[\phi(Y_t, y)|X_t = x\right].$$

For example, we can take $\phi(Y_t, y) = (Y_t - y)_-$ and $\phi(Y_t, y) = (Y_t - y)_+$ for $G_1(y|x) = \mathrm{E}\left[(Y_t - y)_-|X_t = x\right]$ and $G_2(y|x) = \mathrm{E}\left[(Y_t - y)_+|X_t = x\right]$, respectively. Assumptions A1–A5 are assumed with the only changes that the moment conditions should be $\mathrm{E}\left|\phi(Y_t, y)\right|^\delta < \infty$ in Assumption A3.

*Email: zhangfp108@163.com.

†Corresponding author. Email: pszhong@uic.edu.

The local polynomial estimator of $m(y|x) = \mathrm{E}\left[\phi(Y_t, y)|X = x\right]$ is given by

$$\widehat{m}(y|x) = \frac{1}{nh}\sum_{t=1}^{n} W_p\left((X_t - x)/h\right)\phi(Y_t, y),$$

where the kernel weights $W_p(u) = \boldsymbol{e}_1^\top H^{-1}\boldsymbol{S}_n^{-1}H^{-1}(1, u, \ldots, u^p)^\top K(u)$, with the $(p+1)$-dimensional vector $\boldsymbol{e}_1 = (1, 0, \ldots, 0)^\top$ and $H = \mathrm{diag}(1, h, \ldots, h^p)$,

$$\boldsymbol{S}_n = \begin{bmatrix} S_{n,0} & \cdots & S_{n,p} \\ \vdots & \ddots & \vdots \\ S_{n,p} & \cdots & S_{n,2p} \end{bmatrix},$$

with $S_{n,j} = n^{-1}\sum\limits_{t=1}^{n}\left(\frac{X_t - x}{h}\right)^j K_h(X_t - x)$ for $j = 0, \ldots, 2p$, and $K_h(u) = K(u/h)/h$, where $K(\cdot)$ is a kernel function with smoothing bandwidth $h > 0$.

Note that $W_p(u) = \boldsymbol{e}_1^\top \boldsymbol{S}_n^{-1}(1, u, \ldots, u^p)^\top K(u)$ and $\boldsymbol{S}_n = f(x)\boldsymbol{S} + o_P(1)$, by Theorem 1 in Masry and Fan (1997). We can write $W_p(u) = f(x)^{-1}[1 + o_P(1)]K^*(u)$, where $K^*(u) = \boldsymbol{e}_1^\top \boldsymbol{S}^{-1}(1, u, \ldots, u^p)^\top K(u)$, and the $o_p(1)$ part does not depend on u. The equivalent kernel $K^*(u)$ satisfies (Fan and Yao, 2003),

(1) $\int K^*(u)du = 1$,

(2) $\int K^*(u)^2 du = \boldsymbol{e}_1^\top \boldsymbol{S}^{-1}\boldsymbol{S}^*\boldsymbol{S}^{-1}\boldsymbol{e}_1$,

(3) $\int u^{p+1}K^*(u)du = \boldsymbol{e}_1^\top \boldsymbol{S}^{-1}\boldsymbol{c}_p$.

Thus, we can write

$$\begin{aligned} \widehat{m}(y|x) &= \frac{1}{nh}\sum_{t=1}^{n} W_p\left((X_t - x)/h\right)\phi(Y_t, y) \qquad \text{(A.1)} \\ &= f(x)^{-1}\left[1 + o_P(1)\right] n^{-1}\sum_{t=1}^{n}\phi(Y_t, y)K^*\left(\frac{X_t - x}{h}\right)/h, \\ &\equiv f(x)^{-1}\left[1 + o_P(1)\right] n^{-1}\sum_{t=1}^{n} Z_t. \end{aligned}$$

Throughout the paper, for each y, we also assume that the $(p+1)$th partial derivative

$\frac{\partial^{p+1}}{\partial x^{p+1}} m(y|x)$ of $m(y|x)$ at the point x exists and is continuous. By Taylor's expansion, for each y, in the neighborhood of $|X_t - x| \le h$, we have

$$m(y|X_t) = m(y|x) + \cdots + \frac{m^{(p)}(y|x)}{p!}(X_t - x)^p + \frac{m^{(p+1)}(y|x)}{(p+1)!}(X_t - x)^{p+1} + o_p(h^{p+1})$$

$$\equiv \sum_{j=1}^{p} \beta_j(x)(X_t - x)^j + \frac{m^{(p+1)}(y|x)}{(p+1)!}(X_t - x)^{p+1} + o_p(h^{p+1}), \tag{A.2}$$

where $\boldsymbol{\beta}(x) = (\beta_0(x), \ldots, \beta_p(x))^\top$ with $\beta_j(x) = m^{(j)}(y|x)/j!$ for $j = 0, \ldots, p$. Thus, by conditioning on X_t and the Taylor's expansion in (A.2), on can obtain

$$\begin{aligned}
&\mathrm{E}\left[m(y|X_t)K^*\left(\frac{X_t - x}{h}\right)/h\right] \\
&= \mathrm{E}\left[\left\{\sum_{j=1}^{p} \beta_j(x)(X_t - x)^j + \frac{m^{(p+1)}(y|x)}{(p+1)!}(X_t - x)^{p+1} + o_p(h^{p+1})\right\} \boldsymbol{e}_1^\top \boldsymbol{S}^{-1} K\left(\frac{X_t - x}{h}\right)/h\right] \\
&= \int \boldsymbol{e}_1^\top \boldsymbol{S}^{-1} \begin{bmatrix} 1 \\ \vdots \\ \left(\frac{u-x}{h}\right)^p \end{bmatrix} \left[1, \ldots, \left(\frac{u-x}{h}\right)^p\right] H\boldsymbol{\beta}(x) h^{-1} K\left(\frac{u-x}{h}\right) f_X(u)du \\
&\quad + \frac{h^{p+1} m^{(p+1)}(y|x)}{(p+1)!} \int \boldsymbol{e}_1^\top \boldsymbol{S}^{-1} \begin{bmatrix} 1 \\ \vdots \\ \left(\frac{u-x}{h}\right)^p \end{bmatrix} \left(\frac{u-x}{h}\right)^{p+1} h^{-1} K\left(\frac{u-x}{h}\right) f_X(u)du + o_P(h^{p+1}), \\
&= m(y|x) f_X(x) + \frac{\boldsymbol{e}_1^\top \boldsymbol{S}^{-1} \boldsymbol{c}_p m^{(p+1)}(y|x)}{(p+1)!} f_X(x) h^{p+1} + o_P(h^{p+1}).
\end{aligned}$$

To study the asymptotic properties of $\widehat{m}(y|x)$, we consider the centered version of Z_t. We define

$$Z_t^* = \sqrt{h}\left[\phi(Y_t, y) - m(y|X_t)\right] K^*\left(\frac{X_t - x}{h}\right)/h,$$

and

$$Q_n^*(y|x) = n^{-1/2} \sum_{t=1}^{n} Z_t^*.$$

Lemma A.1. *Under Assumptions A1, A2 and A3 with* $\mathrm{E}\left[|\phi(Y,y)|^{\delta}|X=x\right] \leq C_3 < \infty$, *then, as* $n \to \infty$,

(a) $\mathrm{Var}(Z_t^*) \longrightarrow V_0\sigma_\phi^2(y|x)f_X(x)$ *at continuity points of* $f\sigma_\phi$,

(b) $n^{-1}\sum_{i,j=1,i\neq j}^{n} \mathrm{Cov}(Z_j^*, Z_k^*) = o(1)$,

(c) $\mathrm{Var}(Q_n^*(y|x)) \longrightarrow V_0\sigma_\phi^2(y|x)f_X(x)$ *at continuity points of* $f\sigma_\phi$,

where $\sigma_\phi^2(y|x) = \mathrm{Var}(\phi(Y_t,y)|X=x)$ *and* $V_0 = \boldsymbol{e}_1^\top \boldsymbol{S}^{-1}\boldsymbol{S}^*\boldsymbol{S}^{-1}\boldsymbol{e}_1$.

Proof of Lemma A.1.

(a) By conditioning on X_t, we have

$$\begin{aligned}\mathrm{Var}(Z_t^*) = \mathrm{E}[Z_t^{*2}] &= \mathrm{E}\left[\sigma_\phi^2(y|X_t)K^*\left(\frac{X_t-x}{h}\right)^2/h\right]\\ &= \int \sigma_\phi^2(y|x+hu)K^*(u)^2 f_X(x+hu)du\\ &\longrightarrow V_0\sigma_\phi^2(y|x)f_X(x).\end{aligned}$$

(b) By stationarity,

$$n^{-1}\sum_{i,j=1,i\neq j}^{n}\mathrm{Cov}(Z_0^*, Z_l^*) \leq 2\sum_{l=1}^{n-1}(1-l/n)|\mathrm{Cov}(Z_0^*, Z_l^*)|.$$

We decompose the RHS of the above inequality as

$$n^{-1}\sum_{i,j=1,i\neq j}^{n}\mathrm{Cov}(Z_j^*, Z_k^*) \leq 2\left(\sum_{l=1}^{c_n} + \sum_{l=c_n+1}^{n-1}\right)(1-l/n)|\mathrm{Cov}(Z_0^*, Z_l^*)| =: J_1 + J_2,$$

where $c_n \to \infty$ such that $c_n h \to 0$ as $n \to \infty$. Let $x \pm h$ be the interval $[x-h, x+h]$, and $B = \max_{X\in x\pm h}|m(y|X)|$. Note that

$$|\mathrm{Cov}(Z_0^*, Z_l^*)| \leq h\,\mathrm{E}\left|[\phi(Y_0,y)-m(y|X_0)][\phi(Y_l,y)-m(y|X_l)]K^*\left(\frac{X_0-x}{h}\right)K^*\left(\frac{X_l-x}{h}\right)/h^2\right|$$

$$\le h \sup_{X_0, X_l \in x\pm h} \mathrm{E}\left[(|\phi(Y_0,y)|+B)(|\phi(Y_l,y)|+B)|X_0,X_l\right] \mathrm{E}\left|K^*\left(\frac{X_0-x}{h}\right)K^*\left(\frac{X_l-x}{h}\right)/h^2\right|$$
$$\le hA_1 \sup_{X_0, X_l \in x\pm h} \left[\mathrm{E}\left\{(|\phi(Y_0,y)|+B)^2|X_0,X_l\right\}\mathrm{E}\left\{(|\phi(Y_l,y)|+B)^2|X_0,X_l\right\}\right]^{1/2}$$
$$\le Ch.$$

It follows that $J_1 \le Cc_n h = o(1)$ by the choice of c_n.

For J_2, we use Davydov's lemma (Hall and Heyde, 1980, Corollary A2), we have

$$|\operatorname{Cov}(Z_0^*, Z_l^*)| \le 8\,[\alpha(l)]^{1-2/\delta}\left(\mathrm{E}\,|Z_0^*|^\delta\right)^{1/\delta}\left(\mathrm{E}\,|Z_l^*|^\delta\right)^{1/\delta}.$$

By conditioning on X_0 and Assumptions A1–A3, we obtain

$$\begin{aligned}\mathrm{E}\,|Z_0^*|^\delta &\le h^{\delta/2} \sup_{X_0, X_l \in x\pm h} \mathrm{E}\left[(|\phi(Y_0,y)|^\delta+B)|X_0\right]\left[\mathrm{E}\left|h^{-1}K^*(\frac{X_0-x}{h})\right|^\delta\right]\\ &\le h^{\delta/2} \sup_{X_0, X_l \in x\pm h} \mathrm{E}\left[(|\phi(Y_0,y)|^\delta+B)|X_0\right]\left[\int |K^*(u)|^\delta f_X(x+hu)du\right]h^{1-\delta}\\ &\le Ch^{1-\delta/2}\end{aligned}$$

for some $C>0$. Similarly, $\mathrm{E}\,|Z_0^*|^\delta \le Ch^{1-\delta/2}$. Then, it follows that

$$\begin{aligned}J_2 &\le Ch^{2/\delta-1}\sum_{l=c_n+1}^{n}[\alpha(l)]^{1-2/\delta}\\ &\le Ch^{2/\delta-1}c_n^{-a}\sum_{l=c_n+1} l^a[\alpha(l)]^{1-2/\delta} = o(1)\end{aligned}$$

by taking $h^{1-2/\delta}c_n^a = 1$ so that $c_n h \to 0$ holds. Then, part (b) holds.

(c) Note that

$$\operatorname{Var}(Q_n^*(y|x)) = Var(Z_1^*) + n^{-1}\sum_{i,j=1,i\ne j}^{n}\operatorname{Cov}(Z_j^*, Z_k^*).$$

Part (c) follows from parts (a) and (b). □

The following theorem gives the asymptotic normality of $Q_n^*(y|x)$.

Lemma A.2. *Under Assumptions A1–A5 with* $\mathrm{E}\,|\phi(Y,y)|^{\delta} < \infty$*, if* $h = O(n^{-1/(2p+3)})$*, then, as* $n \to \infty$*,*

$$Q_n^* \xrightarrow{\mathcal{D}} N\left(0, f(x)\sigma_\phi^2(y|x)V_0\right)$$

at continuity points of $f\sigma_\phi$*, where* V_0 *and* $\sigma_\phi(y|x)$ *are defined in Lemma A.1.*

Proof of Lemma A.2. We employ the small-block and large-block arguments. Partition $\{1,\ldots,n\}$ into $2k+1$ subsets with large blocks of size $r = r_n$ and small blocks of size $s = s_n$, where $k = [\frac{n}{r_n+s_n}]$. We define the random variables,

$$\eta_j = \sum_{t=j(r+s)+1}^{j(r+s)+r} Z_t^*, \quad ,\xi_j = \sum_{t=j(r+s)+r+1}^{(j+1)(r+s)} Z_t^*, \quad 0 \le j \le k-1,$$

as the sum over the jth large-block and the jth small-block, respectively, and $\zeta_k = \sum_{t=k(r+s)+1}^{n} Z_t^*$ as the sum over the residual block. Then, we can decompose

$$Q_n^*(y|x) = n^{-1/2}\left[\sum_{j=0}^{k-1}\eta_j + \sum_{j=0}^{k-1}\xi_j + \zeta_k\right] \equiv n^{-1/2}\left[Q_n' + Q_n'' + Q_n'''\right].$$

We would show that, as $n \to \infty$,

$$n^{-1}\,\mathrm{E}(Q_n'')^2 \to 0, \quad n^{-1}\,\mathrm{E}(Q_n''')^2 \longrightarrow 0, \tag{A.3}$$

$$\left|\mathrm{E}[\exp(itQ_n')] - \prod_{j=0}^{n}\mathrm{E}[\exp(it\eta_j)]\right| \longrightarrow 0, \tag{A.4}$$

$$n^{-1}\sum_{j=0}^{k-1}\mathrm{E}\,\eta_j^2 \longrightarrow V_0\sigma_\phi^2(y|x)f_X(x), \tag{A.5}$$

$$n^{-1}\sum_{j=0}^{k-1} \mathrm{E}\left[\eta_j^2 I\left\{|\eta_j| > \epsilon\sqrt{n}(V_0\sigma_\phi^2(y|x)f_X(x))^{1/2}\right\}\right] \longrightarrow 0, \tag{A.6}$$

for every $\epsilon > 0$. (A.3) implies that Q_n' and Q_n'' are asymptotic negligible. (A.4) implies that the summands η_j in Q_n' are asymptotic independent, and (A.5) and (A.6) are the standard Lindeberg-Feller conditions for the asymptotic normality of Q_n' under independence.

We first choose the block size. By Assumption A4, there exist constants $b_n \to \infty$ such that

$$b_n s_n = o(\sqrt{nh}), \quad b_n(n/h)^{1/2}\alpha(s_n) \to 0.$$

We define the large blocks size $r_n = [(nh)^{1/2}/b_n]$. It is easy to show that

$$\frac{r_n}{n} \to 0, \quad \frac{s_n}{r_n} \to 0, \quad \frac{r_n}{(nh)^{1/2}} \to 0, \quad \frac{n}{r_n}\alpha_n \to 0. \tag{A.7}$$

To establish (A.3), note that

$$\mathrm{E}(Q_n'')^2 = \sum_{j=0}^{k-1} \mathrm{Var}(\xi_j) + \sum_{t,j=0,t\neq j}^{k-1} \mathrm{Cov}(\xi_t, \xi_j) \equiv F_1 + F_2. \tag{A.8}$$

By stationarity and Lemma A.1,

$$\mathrm{Var}(\xi_j) = s\,\mathrm{Var}(Z_1^*) + 2s\sum_{j=1}^{s-1}(1-\frac{j}{s})\,\mathrm{Cov}(Z_0^*, Z_j^*) = sV_0\sigma_\phi^2(y|x)f_X(x)(1+o(1)). \tag{A.9}$$

Hence, by (A.8) and (A.9),

$$F_1 = O(ks) \sim \frac{ns_n}{r_n + s_n} \sim \frac{ns_n}{r_n} = o(n).$$

Next, with $N_j = j(r+s) + r$,

$$F_2 = \sum_{t,j=0, t \neq j}^{k-1} \sum_{l_1=1}^{s} \sum_{l_2=1}^{s} \mathrm{Cov}(Z^*_{N_j+l_1}, Z^*_{N_j+l_2}), \tag{A.10}$$

and because $t \neq j$, $|N_t - N_j + l_1 - l_2| \geq r$ so that

$$|F_2| \leq 2 \sum_{l_1=1}^{n-r} \sum_{l_2=l_1+r}^{n} |\mathrm{Cov}(Z^*_{l_1}, Z^*_{l2})| \leq 2 \sum_{j=r+1}^{n} |\mathrm{Cov}(Z^*_0, Z^*_j)| = o(n). \tag{A.11}$$

Hence, by (A.8), (A.10) and (A.11), we have, as $n \to \infty$,

$$n^{-1}\,\mathrm{E}(Q''_n)^2 \to 0. \tag{A.12}$$

Using a similar argument,

$$\begin{aligned} n^{-1}\,\mathrm{E}(Q''_n)^2 &\leq \frac{1}{n}[n - k(r+s)]\,\mathrm{Var}(Z^*_0) + 2\sum_{j=1}^{n-1} \mathrm{Cov}(Z^*_0, Z^*_j) \\ &\leq \frac{r_n + s_n}{n} V_0 \sigma^2_\phi(y|x) f_X(x) + o(1) \to 0. \end{aligned} \tag{A.13}$$

Then, (A.3) is proved by (A.12) and (A.13).

To show (A.4), we note that η_j is a function of $\{Z^*_t : t = j(r+s)+1, \ldots, j(r+s)+r\}$, and is thus a function of $\{(Y_{j(r+s)+1}, X_{j(r+s)+1}), \ldots, (Y_{j(r+s)+r}, X_{j(r+s)+r})\}$. Hence, η_j is $\mathcal{F}^{q_j}_{p_j}$-measurable with $p_j = j(r+s)+1$ and $q_j = j(r+s)+1$. With $V_j = \exp(it\eta_j)$, by Volkonskii and Rozanov (1959) Lemma, we have,

$$\left| \mathrm{E}\exp(itQ'_n) - \prod_{j=0}^{k-1} \mathrm{E}\exp(it\eta_j) \right| \leq 16 k_n \alpha(\widetilde{s}_n) \sim \frac{n}{r_n + s_n}\alpha(\widetilde{s}_n) \sim \frac{n}{r_n}\alpha(\widetilde{s}_n), \tag{A.14}$$

where $\widetilde{s}_n = s_n + 1$. Hence, by (A.7), the right hand of (A.14) tends to zero as $n \to \infty$, which establishes (A.4).

To show (A.5), by stationarity and (A.9), with s replaced by r,

$$\operatorname{Var}(\eta_j) = r_n V_0 \sigma_\phi^2(y|x) f_X(x)(1 + o(1)).$$

Hence, $n^{-1} \sum_{j=0}^{r-1} \operatorname{E} \eta_j^2 = \frac{k_n r_n}{n} \sigma_\phi^2(y|x) f_X(x)(1 + o(1)) \to \sigma_\phi^2(y|x) f_X(x)$, establishing (A.5).

To prove (A.6), we need a truncation argument because $\phi(Y, y)$ is not necessarily bounded. For a fixed $L > 0$, we define $c_L(u) = uI(|u| > L)$ and $\phi_L(Y, y) = c_L(\phi(Y, y))$. Let

$$\mu_t^L = h^{1/2} \operatorname{E} \left[\phi_L(Y_t, y) K^*(\frac{X_t - x}{h})/h \right], \quad Z_t^L = h^{1/2} \phi_L(Y_t, y) K^*(\frac{X_t - x}{h})/h - \mu_t^L, \tag{A.15}$$

and

$$Q_n^L(y|x) = n^{-1/2} \sum_{t=1}^n Z_t^L, \quad Q_n^{LC} = n^{-1/2} \sum_{t=1}^n \left[Z_t^* - Z_t^L \right] \tag{A.16}$$

By (A.15) and the boundedness of K, $|Z_t^L| \le C/h^{1/2}$ and thus $|\eta_j^L| \le C \frac{r_n}{h^{1/2}}$, for all $0 \le j \le k-1$, where η_j^L is defined by replacing Z_t^* with Z_t^L in η_j. Thus, $\max_{0 \le j \le k-1} |\eta_j^2| / \sqrt{n} \le C \frac{r_n}{(nh)^{1/2}} \to 0$ by (A.7). Hence, when n is large enough, the set $\{|\eta_j| > \epsilon \sqrt{n} (V_0 \sigma_\phi^2(y|x) f_X(x))^{1/2}\}$ becomes empty. Thus, (A.6) holds for the truncated variables. Hence,

$$n^{-1/2} Q_n^L(y|x) \xrightarrow{\mathcal{D}} N\left(0, f_x(x) \sigma_\phi^L(y|x) V_0\right). \tag{A.17}$$

Define $\Sigma_L(y|x) = f_X(x) \sigma_\phi^L(y|x) V_0$ and $\Sigma(y|x) = f_X(x) \sigma_\phi(y|x) V_0$. To complete the proof, it suffices to show that at first $n \to \infty$ and then $L \to \infty$, we have

$$\operatorname{Var}(Q_n^{LC}) \to 0. \tag{A.18}$$

Indeed,

$$|\operatorname{E} \exp(itQ_n^*) - \exp(-t^2 \Sigma(y|x)/2)|$$

$$\leq |\operatorname{E}\exp(itQ_n^L) - \exp(-t^2\Sigma_L(y|x)/2)| + \operatorname{E}|\exp(itQ_n^L) - 1|$$
$$+ |\exp(-t^2\Sigma_L(y|x)/2) - \exp(-t^2\Sigma(y|x)/2)|.$$

Letting $n \to \infty$, the first term after the inequality sign tends to zero by (A.17) for each $L > 0$; the second term converges to zero by (A.18) at first $n \to \infty$ and then $L \to \infty$; the third term goes to zero as $L \to \infty$ by dominated convergence. It remains to prove (A.18). Note that by (A.16), Q_n^{LC} has the same structure as Q_n^* except that $\phi(Y_t, y)$ is replaced by $\phi(Y_t, y) - \phi_L(Y_t, y)$. Hence, by Lemma A.1, $\lim_{n\to\infty} \operatorname{Var}(Q_n^{LC}) = f_X(x)\left[(\phi(Y_t, y) - \phi_L(Y_t, y))^2 \,|X_t = x\right] \to 0$, as $L \to \infty$ by dominated converges. □

The following theorem provides the asymptotic normality of $\widehat{m}(y|x)$.

Theorem A.3. *Under Assumptions A1, A2 and A3 with* $\operatorname{E}|\phi(Y, y)|^\delta < \infty$*, if* $h = O(n^{-1/(2p+3)})$*, then, as* $n \to \infty$*,*

$$\sqrt{nh}\left[\widehat{m}(y|x) - m(y|x) - \frac{m^{(p+1)}(y|x)\boldsymbol{e}_1^\top \boldsymbol{S}^{-1}\boldsymbol{c}_p}{(p+1)!}h^{p+1}\right] \xrightarrow{\mathcal{D}} N\left(0, \frac{\sigma_\phi^2(y|x)V_0}{f(x)}\right),$$

where $\boldsymbol{e}_1 = (1, \ldots, 0)^\top$, $V_0 = \boldsymbol{e}_1^\top \boldsymbol{S}^{-1}\boldsymbol{S}^*\boldsymbol{S}^{-1}\boldsymbol{e}_1$*, and* $\sigma_\phi^2(y|x)$ *are defined in Lemma A.1.*

Proof of Theorem A.3. The result is directly followed from Lemmas A.1 and A.2. □

To derive the asymptotic normality of $\widehat{\mu}_\tau(Y|x)$, we need to establish the a.s. rates of convergence for $\widehat{m}(y|x) - m(y|x)$ in the follows results.

Assumption A1'.

(a) $\operatorname{E}|\phi(Y_t, y)|^\delta < \infty$ for some $\delta > 2$.

(b) $f_X(x) \leq C_1 < \infty$.

(c) $f_{(X_0,X_l)|(Y_0,Y_l)}(x_0, x_l|y_0, y_l) \leq C_2 < \infty$.

Assumption A2'. The process $\{X_t, Y_t\}$ is stationary α-mixing with the mixing coefficients $\alpha(l)$ satisfying $\sum_{l=1}^{\infty} l^a\alpha(l)^{1-2/\delta} < \infty$ for some $\delta > 2$ and $a > 1 - 2/\delta$, and $\operatorname{E}[|\phi(Y, y)|^\delta|X = x] \leq C_3 < \infty$ for each y.

Assumption A3'. The kernel function satisfies the Lipschitz condition: $|K(u_1) - K(u_2)| \le C_K |u_1 - u_2|$ for some constant $C_K > 0$.

Assumption A4'. $A_f = \inf_x f_X(x) > 0$ and $\mathrm{E}\left[\phi(Y_t, y)|X_t = x\right]$ is a continuous function of y.

Lemma A.4. *Under Assumptions A1'–A4', a compact subset $D \subset \mathbb{R}^2$, let $a_n = \{n \ln n (\ln\ln n)^{1+\eta}\}^{1/\delta}$ for some $\eta > 0$ and $\delta > 2$, and h tends to zero slowly enough such that $r(n) = (nh/\ln n)^{1/2}/a_n \to 0$, as $n \to \infty$. Define $N_n = a_n n^{1/2}/\sqrt{h^3 \ln n}$, and $\psi(n) = \frac{nN_n}{r(n)} \left(\frac{na_n^2}{h \ln n}\right)^{1/4} \alpha(r(n))$. If the strong mixing coefficient $\alpha(n)$ satisfying $\sum_{n=1}^{\infty} \psi(n) < \infty$, then*

$$\sup_{(x,y)\in D} |\widehat{m}(y|x) - m(y|x) - B_n(y|x)| = O\left(\sqrt{\frac{\ln n}{nh}}\right), \quad a.s..$$

Proof of Lemma A.4. We decompose $\widehat{m}(y|x) - m(y|x)$ as

$$\widehat{m}(y|x) - m(y|x) = \widehat{m}(y|x) - \mathrm{E}\,\widehat{m}(y|x) + \mathrm{E}\,\widehat{m}(y|x) - m(y|x).$$

Clearly, the bias term $B_n(y|x) = \mathrm{E}\,\widehat{m}(y|x) - m(y|x) = O_P(h^{p+1})$ is proved in Theorem A.3. It suffices to show that the stochastic term

$$\sup_{(x,y)\in D} |\widehat{m}(y|x) - \mathrm{E}\,\widehat{m}(y|x)| = O\left(\sqrt{\frac{\ln n}{nh}}\right), \quad a.s. \tag{A.19}$$

Since ϕ is not necessarily bounded, we use a truncation technique as in Mack and Silverman (1982) for IID case. Define the truncation version of $\widehat{m}(y|x)$:

$$\widehat{m}_T(y|x) = n^{-1} \sum_{t=1}^{n} W_p\left(\frac{X_t - x}{h}\right) \phi(Y_t, y) I\{|\phi(Y_t, y)| < a_n\}/h.$$

Then,

$$\widehat{m}(y|x) - \mathrm{E}\,\widehat{m}(y|x) = \widehat{m}(y|x) - \widehat{m}_T(y|x) + (\widehat{m}_T(y|x) - \mathrm{E}\,\widehat{m}_T(y|x)) + (\mathrm{E}\,\widehat{m}_T(y|x) - \mathrm{E}\,\widehat{m}(y|x)).$$

we divide the proof in the following three steps.

(i) We first prove $\widehat{m}(y|x) - \widehat{m}_T(y|x) = 0$, a.s.. It follows from Chebychebev inequality, by Assumption A1' (a) and (b),

$$P(|\phi(Y_t, y)| > a_n) \leq a_n^{-\delta} \operatorname{E} |\phi(Y_t, y)|^{\delta}.$$

Since $\sum_{n=1}^{\infty} a_n^{-\delta} < \infty$, by the Borel-Cantelli lemma, $|\phi(Y_t, y)| \leq a_n$, a.s. for all $t \leq n$.

(ii) We would show that, as $n \to \infty$,

$$\sup_{(x,y)\in D} |\operatorname{E}\widehat{m}_T(y|x) - \operatorname{E}\widehat{m}(y|x)| \leq C_1 \operatorname{E} |\phi(Y_1, y)|^{\delta}/a_n^{\delta-1},$$

which tends to zero. Indeed, by stationarity,

$$\begin{aligned}
|\operatorname{E}\widehat{m}_T(y|x) - \operatorname{E}\widehat{m}_T(y|x)| &\leq \operatorname{E}\left[|\phi(Y_t, y)|I\{|\phi(Y_t, y)| > a_n\}\left|h^{-1}W_p\left(\frac{X_t - x}{h}\right)\right|\right] \\
&\leq \frac{1}{a_n^{\delta-1}} \operatorname{E}\left[|\phi(Y_t, y)|^{\delta} \operatorname{E}\left\{\left|h^{-1}W_p\left(\frac{X_t - x}{h}\right)\right| |X_t\right\}\right] \\
&\leq \frac{C_1}{a_n^{\delta-1}} \operatorname{E}\left[|\phi(Y_t, y)|^{\delta}\right] \int |W_p(u)|du.
\end{aligned}$$

(iii) It remains to show that

$$\sup_{(x,y)\in D} |\widehat{m}_T(y|x) - \operatorname{E}\widehat{m}(y|x)| = O\left(\sqrt{\frac{\ln n}{nh}}\right), \quad a.s..$$

Since D is compact, it can be covered by a finite number N of cubes I_k with centers u_k whose sides are of length L (N, I_k, u_k and L depend on the sample size n, but for simplicity we suppress this dependence). We select N in conditions of Lemma A.4 so that $L = C/N_n$. Then,

$$\begin{aligned}
&\sup_{u\in\mathcal{D}} |\widehat{m}_T(y|u) - \operatorname{E}\widehat{m}_T(y|u)| \\
&= \max_{1\leq k\leq N} \sup_{u\in\mathcal{D}\cap I_k} |\widehat{m}_T(y|u) - \operatorname{E}\widehat{m}_T(y|u)|
\end{aligned}$$

$$\le \max_{1\le k\le N} \sup_{u\in\mathcal{D}\cap I_k} |\widehat{m}_T(y|u) - \widehat{m}_T(y|u_k)| + \max_{1\le k\le N} |\widehat{m}_T(y|u_k) - \mathrm{E}\,\widehat{m}_T(y|u_k)|$$

$$+ \max_{1\le k\le N} \sup_{u\in\mathcal{D}\cap I_k} |\mathrm{E}\,\widehat{m}_T(y|u_k) - \mathrm{E}\,\widehat{m}_T(y|u)| =: I_1 + I_2 + I_3.$$

By the definition of $\widehat{m}_T(y|u)$ and Assumption A3', we have

$$|\widehat{m}_T(y|u) - \widehat{m}_T(y|u_k)| \le C a_n h^{-2} |u - u_k|,$$

and hence $I_1 = O(a_n L_n/h^2) = O\left(\sqrt{\frac{\ln n}{nh}}\right)$, a.s.. Similarly, $I_3 = O\left(\sqrt{\frac{\ln n}{nh}}\right)$, a.s.. It suffices to show that

$$I_2 = \max_{1\le k\le N} |\widehat{m}_T(y|u_k) - \mathrm{E}\,\widehat{m}_T(y|u_k)| = O\left(\sqrt{\frac{\ln n}{nh}}\right), \quad \text{a.s..} \tag{A.20}$$

In the following, we estimate $P(I_2 > \epsilon_n)$ and show that for $\epsilon = \epsilon_n = A\sqrt{\frac{\ln n}{nh}}$, where A is a constant to be chosen,

$$\sum_{n=1}^{\infty} P(I_2 > \epsilon_n) \le \sum_{n=1}^{\infty} N_n \sup_{u\in\mathcal{D}\cap I_k} P\left(|\widehat{m}_T(y|u_k) - \mathrm{E}\,\widehat{m}_T(y|u_k)| > \epsilon_n\right) < \infty,$$

thus, by the Borel-Cantelli lemma, (A.20) holds.

Note that

$$\widehat{m}_T(y|x) = f_X(x)^{-1}\left(1 + o_P(1)\right) n^{-1} \sum_{t=1}^{n} \phi(Y_t, y) I\{|\phi(Y_t, y)| < a_n\} K^*\left(\frac{X_t - x}{h}\right)/h.$$

Let

$$Z_t(y|x) = \phi(Y_t, y) I\{|\phi(Y_t, y)| < a_n\} K^*\left(\frac{X_t - x}{h}\right)/h.$$

Then, $\|Z_t(y|x)\|_\infty \le C_1 a_n h^{-1}$. Thus, we can write

$$\widehat{m}_T(y|x) = f(x)^{-1} n^{-1} \sum_{t=1}^{n} Z_t(y|x) + o_P(1).$$

By using the exponential inequality (c.f. Theorem 1.3 of Bosq (1999)) for any $\epsilon > 0$ and each integer $q \in [1, n/2]$,

$$P\left(|\widehat{m}_T(y|u) - \mathrm{E}\,\widehat{m}_T(y|u)| > \epsilon\right) \le 4\exp\left(-\frac{\epsilon^2 q}{8\nu^2(q)}\right) + 22\left(1 + \frac{4C_1 a_n}{h\epsilon}\right)^{1/2} q\alpha(k_n),$$

where $k_n = [\frac{n}{2q}]$, $\nu^2(q) = 2\sigma^2(q)/k_n^2 + C_1 a_n h^{-1}\epsilon/2$ and

$$\sigma^2(q) = \max_{0 \le j \le 2q-1} \mathrm{Var}\left(\phi(Y_{jk_n+1}, y) + \ldots + \phi(Y_{(j+1)k_n+1}, y)\right).$$

By the proof of Lemma A.1, $\sigma^2(q) \le Ck_n/h$ when n is large enough.

By taking $k_n = [\frac{1}{a_n\epsilon}]$ and some simple algebra, we have

$$\sum_{n=1}^{\infty} N_n \exp\left(-\frac{\epsilon^2 q}{8\nu^2(q)}\right) \le C\sum_{n=1}^{\infty} N_n \exp(-\epsilon^2 nh) \le C\sum_{n=1}^{\infty} \frac{N_n}{n^{A^2}}.$$

By setting A large enough, we can ensure that N_n/n^{A^2} is summable. On the other hand, some algebraic manipulations show that

$$\sum_{n=1}^{\infty} N_n \left(1 + \frac{4a_n}{h\epsilon}\right)^{1/2} q\alpha(k_n) \le C\sum_{n=1}^{\infty} \psi(n) < \infty,$$

by the assumption. Thus,

$$\sum_{n=1}^{\infty} P(I_2 > \epsilon) \le \sum_{n=1}^{\infty} N_n \left[4\exp\left(-\frac{\epsilon^2 q}{8\nu^2(q)}\right) + 22\left(1 + \frac{4a_n}{h\epsilon}\right)^{1/2} q\alpha(k_n)\right] < \infty.$$

Then, (A.20) holds by the Borel-Cantelli lemma. □

The following result provides the local oscillation and modulli of continuity of $\widehat{G}(y|x)$.

Lemma A.5. *Under Assumptions A1'–A4', we have*

$$\sup_x \sup_{|\Delta y| \le c\sqrt{\ln n/nh}} |\omega_n(\Delta y|x)| = O\left(\left(\frac{\ln n}{nh}\right)^{3/4}\right), \quad a.s.,$$

where $\omega_n(\Delta y|x) = \widehat{G}(y + \Delta y|x) - \widehat{G}(y|x) - G(y + \Delta y|x) + G(y|x)$.

Proof of Lemma A.5. Note that

$$\widehat{G}(y|x) - G(y|x) = \frac{1}{\widehat{G}_2(y|x)}\left[\widehat{G}_1(y|x) - G_1(y|x)\right] - \frac{\widehat{G}(y|x)}{\widehat{G}_2(y|x)}\left[\widehat{G}_2(y|x) - G_2(y|x)\right].$$

Thus,

$$\begin{aligned}
&\omega_n(\Delta y|x)\\
&= \frac{1}{\widehat{G}_2(y+\Delta y|x)}\left[\widehat{G}_1(y+\Delta y|x) - G_1(y+\Delta y|x)\right] - \frac{1}{\widehat{G}_2(y|x)}\left[\widehat{G}_1(y|x) - G_1(y|x)\right]\\
&\quad - \left\{\frac{G(y+\Delta y|x)}{\widehat{G}_2(y+\Delta y|x)}\left[\widehat{G}_2(y+\Delta y|x) - G_2(y+\Delta y|x)\right] - \frac{G(y|x)}{\widehat{G}_2(y|x)}\left[\widehat{G}_2(y+\Delta y|x) - G_2(y|x)\right]\right\}\\
&=: \omega_{n1}(\Delta y|x) - \omega_{n2}(\Delta y|x).
\end{aligned}$$

We first consider $\omega_{n1}(\Delta y|x)$. By Lemma A.4, we have $\sup_{(x,y)\in D}|\widehat{G}_2(y|x) - G_2(y|x)| = O\left(\sqrt{\frac{\ln n}{nh}}\right) + O(h^{p+1})$, a.s.. Noting that

$$\omega_{n1}(\Delta y|x) = \frac{\widehat{G}_2(y|x)[\widehat{G}_1(y+\Delta y|x) - G_1(y+\Delta y|x)] - \widehat{G}_2(y+\Delta y|x)[\widehat{G}_1(y|x) - G_1(y|x)]}{\widehat{G}_2(y+\Delta y|x)\widehat{G}_2(y|x)}.$$

The denominator of $\omega_{n1}(\Delta y|x)$ is $G_(y|x)^2\left[1 + O\left(\sqrt{\frac{\ln n}{nh}}\right) + O(h^{p+1})\right]$ almost surely. Hence, we only need to work with its numerator. Further, we could check with bias term,

$$\begin{aligned}
&\widehat{G}_2(y|x)[\widehat{G}_1(y+\Delta y|x) - G_1(y+\Delta y|x)] - \widehat{G}_2(y+\Delta y|x)[\widehat{G}_1(y|x) - G_1(y|x)]\\
&= [\widehat{G}_2(y|x) - \widehat{G}_2(y+\Delta y|x)][\widehat{G}_1(y+\Delta y|x) - \widehat{G}_1(y+\Delta y|x)]\\
&\quad + \widehat{G}_2(y+\Delta y|x)\left[\widehat{G}_1(y+\Delta y|x) - G_1(y+\Delta y|x) - \widehat{G}_1(y|x) + G_1(y|x)\right].
\end{aligned}$$

Without loss of generality, we assume $\Delta y > 0$ and $\Delta y = O\left(\sqrt{\frac{\ln n}{nh}}\right)$. Similar to the proof of Lemma A.1, some tedious calculations can show that $|\widehat{G}_2(y|x) - \widehat{G}_2(y+\Delta y|x)| = O_P(\Delta y) = O\left(\sqrt{\frac{\ln n}{nh}}\right)$ almost surely, and $|\widehat{G}_1(y+\Delta y|x) - \widehat{G}_1(y+\Delta y|x)| = O(h^{p+1})$

almost surely. Thus, almost surely,

$$|[\widehat{G}_2(y|x) - \widehat{G}_2(y+\Delta y|x)][\widehat{G}_1(y+\Delta y|x) - \widehat{G}_1(y+\Delta y|x)]| = O\left(\sqrt{\frac{\ln n}{nh}} h^{p+1}\right) = O\left(\left(\frac{\ln n}{nh}\right)^{3/4}\right),$$

if $h = O\left(\left(\frac{\ln n}{nh}\right)^{1/(4p+5)}\right)$.

Now, we need to check

$$\Delta_1(\Delta y|x) = \widehat{G}_1(y+\Delta y|x) - G_1(y+\Delta y|x) - \widehat{G}_1(y|x) + G_1(y|x).$$

We decompose it as

$$\begin{aligned}\Delta_1(\Delta y|x) &= \widehat{G}_1(y+\Delta y|x) - \widehat{G}_1(y|x) - \mathrm{E}\left[\widehat{G}_1(y+\Delta y|x) - \widehat{G}_1(y|x)\right] \\ &\quad + \mathrm{E}\left[\widehat{G}_1(y+\Delta y|x) - \widehat{G}_1(y|x)\right] - [G_1(y+\Delta y|x) - G_1(y|x)] \\ &=: \Delta_{11}(\Delta y|x) + \Delta_{12}(\Delta y|x).\end{aligned}$$

For the bias component $\Delta_{12}(\Delta y|x)$, we can take $\phi(Y_t, y) = (Y_t - y)_- - (Y_t - y - \Delta y)_- = -(Y_t - y - \Delta y)I(y \le Y_t < y + \Delta y) + \Delta y I(Y_t \le y)$. Similar to the arguments in the proof of Lemma A.1, we can readily obtain, $\Delta_{12}(y|x) = O(\Delta y h^{p+1}) = O\left(\left(\frac{\ln n}{nh}\right)^{3/4}\right)$ almost surely if $h = O\left(\left(\frac{\ln n}{nh}\right)^{1/(4p+5)}\right)$.

For the stochastic component $\Delta_{11}(\Delta y|x)$,

$$\begin{aligned}&|\Delta_{11}(\Delta y|x)| \\ &= \left| \int\int h^{-1} W_p\left(\frac{X_t - x}{h}\right) \phi(Y_t, y) d\left[\mathcal{C}_n(F_X(X_t), F_Y(Y_t)) - \mathcal{C}(F_X(X_t), F_Y(Y_t))\right] \right| \\ &\le C\Delta y \int\int d\left|\mathcal{C}_n(F_X(X_t), F_Y(Y_t)) - \mathcal{C}(F_X(X_t), F_Y(Y_t))\right|,\end{aligned}$$

where $\mathcal{C}_n$ and $\mathcal{C}$ are the empirical and population copula function between (Y, X), respectively. The double integral term corresponds the multivariate local oscillation of empirical process within a shrinkage rectangle studied by Stute (1984a). By Theorem 1.5 or Theorem 3.1 in Stute (1984b) and existence and boundedness of copula density, we

have

$$\begin{aligned}
\sup_{(x,y)\in D} |\Delta_{11}(\Delta y|x)| &= C\Delta y \cdot O\left(\sqrt{\frac{\log(h\epsilon)^{-1}}{nh\epsilon}}\right) \text{ a.s.} \\
&= O\left(\epsilon\sqrt{\frac{\log(h\epsilon)^{-1}}{nh\epsilon}}\right) = O\left(\frac{1}{h}\frac{\sqrt{h}\sqrt{\ln n}}{\sqrt{n}}\left(\frac{\ln n}{nh}\right)^{1/4}\right) \text{ a.s.} \\
&= O\left(\left(\frac{\ln n}{nh}\right)^{3/4}\right) \text{ a.s.}
\end{aligned}$$

Similarly, we can show that $\sup_{(x,y)\in D} |\Delta_{n2}(\Delta y|x)| = O\left(\left(\frac{\ln n}{nh}\right)^{3/4}\right)$ almost surely. The proof is completed. □

Appendix B Proofs of Main Results in Section 2

By applying Theorem A.3, we now prove the results in Theorem 1.

Proof of Theorem 1. Note that we can take $\phi(Y_t, y) = (Y_t - y)_-$ and $\phi(Y_t, y) = (Y_t - y)_+$ for $G_1(y|x) = \mathrm{E}\left[(Y_t - y)_-|X = x\right]$ and $G_2(y|x) = \mathrm{E}\left[(Y_t - y)_+|X = x\right]$, respectively. By applying Theorem A.3, we have

$$\begin{aligned}
\sqrt{nh}\left[\widehat{G}_1(y|x) - G_1(y|x) - B_1(y|x)h^{p+1}\right] &\xrightarrow{\mathcal{D}} N\left(0, \frac{V_0\sigma_1^2(y|x)}{f(x)}\right), \\
\sqrt{nh}\left[\widehat{G}_2(y|x) - G_2(y|x) - B_2(y|x)h^{p+1}\right] &\xrightarrow{\mathcal{D}} N\left(0, \frac{V_0\sigma_2^2(y|x)}{f(x)}\right),
\end{aligned}$$

where $B_k(y|x) = \frac{G_k^{(p+1)}(y|x)\boldsymbol{e}_1^\top \boldsymbol{S}^{-1}\boldsymbol{c}_p}{(p+1)!}$ for $k = 1, 2$ are the bias terms, $\sigma_1^2(y|x) = \mathrm{Var}((Y - y)_-|X = x)$ and $\sigma_2^2(y|x) = \mathrm{Var}((Y - y)_+|X = x)$ are the variance terms. Observe that

$$\widehat{G}(y|x) - G(y|x) = \frac{1}{\widehat{G}_2(y|x)}\left[\widehat{G}_1(y|x) - G_1(y|x)\right] - \frac{G(y|x)}{\widehat{G}_2(y|x)}\left[\widehat{G}_2(y|x) - G_2(y|x)\right].$$

We will show that

$$\mathrm{Cov}\left(\widehat{G}_1(y|x), \widehat{G}_2(y|x)\right) = o\left(\frac{1}{nh}\right). \tag{B.1}$$

From this, it follows that

$$\sqrt{nh}\left[\widehat{G}(y|x) - G(y|x) - B_G(y|x)\right] \xrightarrow{\mathcal{D}} N\left(0, \Sigma_G(y|x)\right),$$

where

$$\begin{aligned}
B_G(y|x) &= \left[\frac{1}{G_2(y|x)}B_1(y|x) - \frac{G(y|x)}{G_2(y|x)}B_2(y|x)\right]h^{p+1}, \\
\Sigma_G(y|x) &= \frac{V_0}{f(x)}\left[\frac{\sigma_1^2(y|x)}{G_2(y|x)^2} + \frac{G(y|x)^2\sigma_2^2(y|x)}{G_2(y|x)^2}\right].
\end{aligned}$$

It remains to show (B.1). First, we can write

$$\begin{aligned}
\widehat{G}_1(y|x) &= \frac{1}{nh}\sum_{t=1}^{n} W_p\left((X_t - x)/h\right)(Y_t - y)_- \\
&= f(x)^{-1}\left[1 + o_P(1)\right] n^{-1}\sum_{t=1}^{n} K^*\left(\frac{X_t - x}{h}\right)(Y_t - y)_-/h, \\
&\equiv f(x)^{-1}\left[1 + o_P(1)\right] n^{-1}\sum_{t=1}^{n} Z_{1t},
\end{aligned}$$

and

$$\begin{aligned}
\widehat{G}_2(y|x) &= \frac{1}{nh}\sum_{t=1}^{n} W_p\left((X_t - x)/h\right)(Y_t - y)_+ \\
&= f(x)^{-1}\left[1 + o_P(1)\right] n^{-1}\sum_{t=1}^{n} K^*\left(\frac{X_t - x}{h}\right)(Y_t - y)_+/h, \\
&\equiv f(x)^{-1}\left[1 + o_P(1)\right] n^{-1}\sum_{t=1}^{n} Z_{2t}.
\end{aligned}$$

By stationarity, we have

$$\begin{aligned}
\operatorname{Cov}\left(n^{-1}\sum_{t=1}^{n} Z_{1t}, n^{-1}\sum_{t=1}^{n} Z_{2t}\right) &= n^{-1}\operatorname{Cov}(Z_{11}, Z_{21}) + \frac{2}{n}\sum_{l=1}^{n-1}(1 - \frac{l}{n})\operatorname{Cov}(Z_{11}, Z_{2l}) \\
&\equiv I + II.
\end{aligned}$$

For I, by conditioning on X_1 and Assumptions A2, A3,

$$
\begin{aligned}
I &= \frac{1}{nh^2} \mathrm{E} \left[-K^* \left(\frac{X_1 - x}{h} \right)^2 (Y_1 - y)_- (Y_1 - y)_+ \right] \\
&\quad - \frac{1}{nh^2} \mathrm{E} \left[-K^* \left(\frac{X_1 - x}{h} \right) (Y_1 - y)_- \right] \mathrm{E} \left[K^* \left(\frac{X_1 - x}{h} \right) (Y_1 - y)_+ \right] \\
&= \frac{1}{n} G_1(y|x) G_2(y|x) f(x)^2 = O(n^{-1}) = o\left(\frac{1}{nh} \right).
\end{aligned}
$$

For II, let $d_n \to \infty$ be a sequences of integers such that $d_n h \to \infty$. Define

$$
J_1 = \sum_{l=1}^{d_n - 1} |\operatorname{Cov}(Z_{11}, Z_{l2})|, \quad J_2 = \sum_{l=d_n}^{n-1} |\operatorname{Cov}(Z_{11}, Z_{2l})|.
$$

Let $B_1 = \max_{X \in x \pm h} |G_1(y|x)|$ and $B_2 = \max_{X \in x \pm h} |G_2(y|x)|$. By conditioning on (X_1, X_l) and using Assumption A3,

$$
\begin{aligned}
&|\operatorname{Cov}(Z_{11}, Z_{l2})| \\
&= \left| \mathrm{E} \left[\left(-(Y_1 - y)_- - G_1(y|x) \right) \left((Y_l - y)_+ - G_2(y|x) \right) K^* \left(\frac{X_1 - x}{h} \right) K^* \left(\frac{X_l - x}{h} \right) \right] \right| \\
&\le \sup_{X_1, X_l \in x \pm h} \mathrm{E} \left[\left(|(Y_1 - y)_-| + B_1 \right) \left(|(Y_1 - y)_+| + B_2 \right) |X_1, X_l \right] \mathrm{E} \left| K^* \left(\frac{X_1 - x}{h} \right) K^* \left(\frac{X_l - x}{h} \right) \right| \\
&\le \sup_{X_1, X_l \in x \pm h} \left(\mathrm{E} \left[\left(|(Y_1 - y)_-| + B_1 \right)^2 |X_1, X_l \right] \mathrm{E} \left[\left(|(Y_l - y)_+| + B_2 \right)^2 |X_1, X_l \right] \right)^{1/2} \\
&\quad \times A_1 \left[\int \left| K^* \left(\frac{u - x}{h} \right) \right| du \right]^2 \le C.
\end{aligned}
$$

It follows that $J_1 \le d_n C = o(1/h)$.

We now consider J_2. By Davydov's lemma (Hall and Heyde, 1980, Corollary A2), we have

$$
|\operatorname{Cov}(Z_{11}, Z_{l2})| \le 8 \left[\alpha(l) \right]^{1 - 2/\delta} \left(\mathrm{E} \, |Z_{11}|^\delta \right)^{1/\delta} \left(\mathrm{E} \, |Z_{l2}|^\delta \right)^{1/\delta}.
$$

By conditioning on X_1 and Assumptions A1, A2 and A3, we obtain

$$\begin{aligned}
\mathrm{E}\,|Z_{11}|^{\delta} &\le B_1^{\delta}\,\mathrm{E}\left[\left|h^{-1}K^{*}(\frac{X_1-x}{h})\right|^{\delta}\right] \\
&\le B_1^{\delta}\int h^{-\delta}\left|\boldsymbol{e}_1^{\top}\boldsymbol{S}^{-1}(1,\ldots,(\frac{u-x}{h})^{p})^{\top}K\left(\frac{u-x}{h}\right)\right|^{\delta} f_X(u)du \\
&\le B_1^{\delta}h^{-\delta+1}\sup_{v\in x\pm h} f_X(v)\int\left|\boldsymbol{e}_1^{\top}\boldsymbol{S}^{-1}(1,\ldots,u^{p})^{\top}K(u)\right|^{\delta}du, \\
&\le Ch^{-\delta+1},
\end{aligned}$$

for some $C>0$. Similarly, we can show $\mathrm{E}\,|Z_{11}|^{\delta}\le Ch^{-\delta+1}$. Thus, combining the last three inequalities leads to

$$\begin{aligned}
J_2 &\le 8C^{2/\delta}h^{2/\delta-2}\sum_{l=d_n}^{n-1}\alpha(l)^{1-2/\delta} \\
&\le 8C^{2/\delta}h^{2/\delta-2}d_n^{a}\sum_{l=d_n}^{n-1}l^{a}\alpha(l)^{1-2/\delta}=o(1/h),
\end{aligned}$$

by taking $h^{1-2/\delta}d_n^{a}=1$ so that $d_nh\to 0$. Therefore, we conclude that $J_2=2/n\sum_{l=d_n}^{n-1}|\operatorname{Cov}(Z_{11},Z_{l2})|=o(1/nh)$. This implies that (B.1) holds. □

Proof of Theorem 2. Note that

$$\begin{aligned}
&G\left(\widehat{\mu}_{\tau}(x)|x\right)-G(\mu_{\tau}(x)|x) \\
=&-\left[\widehat{G}\left(\widehat{\mu}_{\tau}(x)\right)-G\left(\widehat{\mu}_{\tau}(x)\right)\right] \\
=&-\left[\widehat{G}\left(\mu_{\tau}(x)|x\right)-G\left(\mu_{\tau}(x)|x\right)\right] \\
&-\left[\widehat{G}\left(\widehat{\mu}_{\tau}(x)|x\right)-G\left(\widehat{\mu}_{\tau}(x)|x\right)-\widehat{G}\left(\mu_{\tau}(x)|x\right)+G\left(\mu_{\tau}(x)|x\right)\right] \\
=:&\ I_1+I_2.
\end{aligned}$$

By Lemma A.5, we have $\sup_{(x,y)\in D}|\sqrt{nh}I_2|=O_P\left(\sqrt{nh}\left(\frac{\ln n}{nh}\right)^{3/4}\right)=o_P(1)$. By Taylor's

expansion on the left hand side,

$$G(\widehat{\mu}_\tau(x)|x) - G(\mu_\tau(x)|x) = G'(\mu_\tau(x)|x)\,[\widehat{\mu}_\tau(x) - \mu_\tau(x)] + o_P(1).$$

Thus, we have

$$\begin{aligned}
&\sqrt{nh}\,[\widehat{\mu}_\tau(x) - \mu_\tau(x)] \\
&= -[G'(\mu_\tau(x)|x)]^{-1}\sqrt{nh}\left[\widehat{G}(\mu_\tau(x)) - G(\mu_\tau(x))\right] + o_P(1) \\
&= -[G'(\mu_\tau(x)|x)]^{-1}\sqrt{nh}\left[\widehat{G}(\mu_\tau(x)) - G(\mu_\tau(x)) - B_G(\mu_\tau(x)|x)\right] \\
&\quad - [G'(\mu_\tau(x)|x)]^{-1}\sqrt{nh}B_G(\mu_\tau(x)|x) + o_P(1).
\end{aligned}$$

Thus, we can rearrange the above representation as

$$\begin{aligned}
&\sqrt{nh}\,[\widehat{\mu}_\tau(x) - \mu_\tau(x) - B_\tau(x)] \\
&= -[G'(\mu_\tau(x)|x)]^{-1}\sqrt{nh}\left[\widehat{G}(\mu_\tau(x)) - G(\mu_\tau(x)) - B_G(\mu_\tau(x)|x)\right] + o_P(1) \\
&\xrightarrow{\mathcal{D}} N\left(0, \Sigma_\tau(x)\right),
\end{aligned}$$

where the bias is $B_\tau(x) = -[G'(\mu_\tau(x)|x)]^{-1}B_G(\mu_\tau(x)|x)$, and the asymptotic variance is $\Sigma_\tau(x) = [G'(\mu_\tau(x)|x)]^{-2}\Sigma_G\left(\mu_\tau(x)|x\right)$. □

Proof of Theorem 3. Recall that

$$\begin{aligned}
\widetilde{\Sigma}_\tau(x) &= \frac{V_0}{f_X(x)}\frac{\mathrm{E}\left[w_\tau^2(Y_t - \mu_\tau(x))(Y_t - \mu_\tau(x))^2|X_t = x\right]}{\left[\mathrm{E}\left\{w_\tau(Y_t - \mu_\tau(x))|X_t = x\right\}\right]^2}, \\
\Sigma_\tau(x) &= \frac{V_0}{f_X(x)}\left[\frac{F(\mu_\tau(x)|x)G_2(\mu_\tau(x)|x) + (1 - F(\mu_\tau(x)|x))G_1(\mu_\tau(x)|x)}{G_2^2(\mu_\tau(x)|x)}\right]^{-2} \\
&\quad \times \frac{\sigma_1^2(\mu_\tau(x)|x) + G(\mu_\tau(x))^2\sigma_2^2(\mu_\tau(x)|x)}{G_2(\mu_\tau(x)|x)^2}.
\end{aligned}$$

Note that $\mathrm{E}\left[w_\tau(Y_t - \mu_\tau(x))|X_t = x\right] = (1-\tau)F(\mu_\tau(x)|x) + \tau(1 - F(\mu_\tau(x)|x))$, and

$$G(\mu_\tau(x)|x) = \frac{G_2(\mu_\tau(x)|x)}{G_1(\mu_\tau(x)|x)}.$$

Some simple algebraic calculations can show that

$$\Sigma_\tau(x) = \frac{V_0}{f_X(x)} \frac{(1-\tau)^2\sigma_1^2(\mu_\tau(x)|x) + \tau^2\sigma_2^2(\mu_\tau(x)|x)}{\left[\mathrm{E}\left\{w_\tau(Y_t - \mu_\tau(x))|X_t = x\right\}\right]^2}.$$

Then, to show that $\Sigma(x) \le \widetilde{\Sigma}_\tau(x)$, it suffices to show that

$$\begin{aligned} &(1-\tau)^2\sigma_1^2(\mu_\tau(x)|x) + \tau^2\sigma_2^2(\mu_\tau(x)|x) \\ &\le \mathrm{E}\left[w_\tau^2(Y_t - \mu_\tau(x))(Y_t - \mu_\tau(x))^2|X_t = x\right]. \end{aligned} \tag{B.2}$$

Indeed, one can easily show that

$$\mathrm{Var}[g(Y_t)|X_t = x] = \mathrm{Var}\left[\int_0^{Y_t} g'(s)ds|X_t = x\right] \le \mathrm{E}\left[\int_0^{Y_t} g'(s)ds|X_t = x\right]^2,$$

where the first equality holds because $g(y)$ and $\int_0^x g'(s)ds$ differ by a constant. By using this fact, we can easily show that

$$\begin{aligned} \sigma_1^2(\mu_\tau(x)|x) &= \mathrm{Var}\left[(Y_t - \mu_\tau(x))_- \,|X_t = x\right] \le \mathrm{E}\left[(Y_t - \mu_\tau(x))_-^2 \,|X_t = x\right], \\ \sigma_2^2(\mu_\tau(x)|x) &= \mathrm{Var}\left[(Y_t - \mu_\tau(x))_+ \,|X_t = x\right] \le \mathrm{E}\left[(Y_t - \mu_\tau(x))_+^2 \,|X_t = x\right]. \end{aligned} \tag{B.3}$$

By the definition of $w_\tau(\cdot)$, we have

$$\begin{aligned} &\mathrm{E}\left[w_\tau^2(Y_t - \mu_\tau(x))\,(Y_t - \mu_\tau(x))^2\,|X_t = x\right] \\ &= (1-\tau)^2\,\mathrm{E}\left[(Y_t - \mu_\tau(x))_-^2\,|X_t = x\right] + \tau^2\,\mathrm{E}\left[(Y_t - \mu_\tau(x))_+^2\,|X_t = x\right]. \end{aligned} \tag{B.4}$$

By (B.3) and (B.4), then (B.2) holds. Clearly, the equality in (B.2) holds if and only if Y_t's are constants. □

Appendix C Detailed Bias Comparison of Two Estimators

We aim at comparing the asymptotic biases of the proposed estimator $\widehat{\mu}_\tau(x)$ and the IWLS estimator $\widetilde{\mu}_\tau(x)$. By a simple manipulation, the asymptotic bias of $\widehat{\mu}_\tau(x)$ is exactly

$$B_\tau(x) = \frac{\tau G_2^{(p+1)}\left\{\mu_\tau(x)|x\right\} - (1-\tau)G_1^{(p+1)}\left\{\mu_\tau(x)|x\right\}}{\tau\left[1 - F\left\{\mu_\tau(x)|x\right\}\right] + (1-\tau)F\left\{\mu_\tau(x)|x\right\}} \frac{\boldsymbol{e}_1^\top \boldsymbol{S}^{-1}\boldsymbol{c}_p}{(p+1)!} h^{p+1}.$$

Recall that the asymptotic bias of $\widetilde{\mu}_\tau(x)$ is

$$\widetilde{B}_\tau(x) = \mu_\tau^{(p+1)}(x) \frac{\boldsymbol{e}_1^\top \boldsymbol{S}^{-1}\boldsymbol{c}_p}{(p+1)!} h^{p+1}.$$

Then, it is sufficient to compare the first part of $B_\tau(x)$ with the first part of $B_\tau(x)$, which are given by, respectively,

$$\frac{\tau G_2^{(p+1)}\left\{\mu_\tau(x)\right\} - (1-\tau)G_1^{(p+1)}\left\{\mu_\tau(x)|x\right\}}{\tau\left[1 - F\left\{\mu_\tau(x)|x\right\}\right] + (1-\tau)F\left\{\mu_\tau(x)|x\right\}} \quad \text{and} \quad \mu_\tau^{(p+1)}(x).$$

Recall that $m^{(j)}(y|x) = \frac{\partial^j}{\partial x^j} m(y|x)$ are jth order partial derivative of a generic function $m(\cdot|x)$ with respect to x. To simplify our notations, we denote $\mu_\tau = \mu_\tau(x)$, $F(\mu_\tau) = F(\mu_\tau|x)$ and $m(\mu_\tau) = m(\mu_\tau|x)$, which are functions of x. Since μ_τ satisfies the first order condition:

$$(1-\tau)G_1(\mu_\tau|x) = \tau G_2(\mu_\tau|x),$$

then by taking derivative with respect to x, we have

$$(1-\tau)G_1'(\mu_\tau)\mu_\tau^{(1)} + (1-\tau)G_1^{(1)}(\mu_\tau) = \tau G_2'(\mu_\tau)\mu_\tau^{(1)} + \tau G_2^{(1)}(\mu_\tau).$$

Recall that $G_1'(y|x) := \frac{d}{dy} G_1(y|x) = F(y|x)$ and $G_2'(y|x) := \frac{d}{dy} G_2(y|x) = F(y|x) - 1$ are first order derivatives with respect to y.

Then, for $p = 0$, by some algebra manipulations, we have

$$[(1-\tau)F(\mu_\tau) + \tau(1 - F(\mu_\tau))]\mu_\tau^{(1)} = \tau G_2^{(1)}(\mu_\tau) - (1-\tau)G_1^{(1)}(\mu_\tau). \tag{C.1}$$

This implies that

$$\mu_\tau^{(1)} = \frac{\tau G_2^{(1)}(\mu_\tau) - (1-\tau)G_1^{(1)}(\mu_\tau)}{[(1-\tau)F(\mu_\tau) + \tau(1-F(\mu_\tau)]}$$

and hence, $B_\tau(x) = \widetilde{B}_\tau(x)$ when $p = 0$.

Next, we consider the case of $p = 1$. By taking derivative of (C.1) with respect to x, we have

$$\begin{aligned}
&[(1-\tau)F'(\mu_\tau)\mu_\tau^{(1)} + (1-\tau)F^{(1)}(\mu_\tau) - \tau F'(\mu_\tau)\mu_\tau^{(1)} - \tau F^{(1)}(\mu_\tau)]\mu_\tau^{(1)} \\
&+ [(1-\tau)F(\mu_\tau) + \tau(1-F(\mu_\tau)]\mu_\tau^{(2)} \\
= &\tau G_2^{(2)}(\mu_\tau) - (1-\tau)G_1^{(2)}(\mu_\tau) + \tau G_2^{'(1)}(\mu_\tau)\mu_\tau^{(1)} - (1-\tau)G_1^{'(1)}(\mu_\tau)\mu_\tau^{(1)}.
\end{aligned}$$

Note that $G_1^{'(1)}(y|x) := \frac{d^2}{dydx}G_1(y|x) = \frac{d}{dx}F(y|x) = F^{(1)}(y|x)$ and $G_2^{'(1)}(y|x) := \frac{d^2}{dydx}G_2(y|x) = \frac{d}{dx}F(y|x) = F^{(1)}(y|x)$. Some algebra manipulations show that

$$\begin{aligned}
&[(1-\tau)F(\mu_\tau) + \tau(1-F(\mu_\tau)]\,\mu_\tau^{(2)} - \left[\tau G_2^{(2)}(\mu_\tau) - (1-\tau)G_1^{(2)}(\mu_\tau)\right] \\
= &(2\tau-1)\left[f(\mu_\tau)\left(\mu_\tau^{(1)}\right)^2 + 2F(\mu_\tau)\mu_\tau^{(1)}\right].
\end{aligned} \tag{C.2}$$

Thus, we have

$$\widetilde{B}_\tau(x) - B_\tau(x) = \frac{(2\tau-1)\left[f(\mu_\tau)\left(\mu_\tau^{(1)}\right)^2 + 2F(\mu_\tau)\mu_\tau^{(1)}\right]}{\tau\left[1-F\left(\mu_\tau\right)\right] + (1-\tau)F\left(\mu_\tau\right)}\frac{\boldsymbol{e}_1^\top \boldsymbol{S}^{-1}\boldsymbol{c}_1}{2!}h^2.$$

Clearly, the sign of $\widetilde{B}_\tau(x) - B_\tau(x)$ mainly depends on $(2\tau-1)\left[f(\mu_\tau)\left(\mu_\tau^{(1)}\right)^2 + 2F(\mu_\tau)\mu_\tau^{(1)}\right]$ and the sign of $\boldsymbol{e}_1^\top \boldsymbol{S}^{-1}\boldsymbol{c}_1$. Interestingly, when $\tau = 0.5$, $\widetilde{B}_\tau(x) = B_\tau(x)$.

Similarly, for $p = 2$, by taking derivative of (C.3) with respect to x, we have

$$\begin{aligned}
&[(1-\tau)F(\mu_\tau) + \tau(1-F(\mu_\tau)]\,\mu_\tau^{(3)} - \left[\tau G_2^{(3)}(\mu_\tau) - (1-\tau)G_1^{(3)}(\mu_\tau)\right] \\
&+ \left[(1-\tau)f(\mu_\tau)\mu_\tau^{(1)} + (1-\tau)F^{(1)}(\mu_\tau) - \tau f(\mu_\tau)\mu_\tau^{(1)} - \tau F^{(1)}(\mu_\tau)\right]\mu_\tau^{(2)} \\
&- \left[\tau G_2^{'(2)}(\mu_\tau)\mu_\tau^{(1)} - (1-\tau)G_1^{'(2)}(\mu_\tau)\mu_\tau^{(1)}\right]
\end{aligned}$$

$$= (2\tau - 1)\left[f'(\mu_\tau)\left(\mu_\tau^{(1)}\right)^3 + f^{(1)}(\mu_\tau)\left(\mu_\tau^{(1)}\right)^2 + 2f(\mu_\tau)\mu_\tau^{(1)}\mu_\tau^{(2)} \right.$$
$$\left. +2f(\mu_\tau)\left(\mu_\tau^{(1)}\right)^2 + 2F^{(1)}(\mu_\tau)\mu_\tau^{(1)} + 2F(\mu_\tau)\mu_\tau^{(2)}\right].$$

Then, it is not difficult to see that

$$\begin{aligned}
&[(1-\tau)F(\mu_\tau) + \tau(1 - F(\mu_\tau)]\,\mu_\tau^{(3)} - \left[\tau G_2^{(3)}(\mu_\tau) - (1-\tau)G_1^{(3)}(\mu_\tau)\right] \\
= (2\tau - 1)&\left[f'(\mu_\tau)\left(\mu_\tau^{(1)}\right)^3 + [f^{(1)}(\mu_\tau) + 2f(\mu_\tau)]\left(\mu_\tau^{(1)}\right)^2 + 3f(\mu_\tau)\mu_\tau^{(1)}\mu_\tau^{(2)} \right. \\
&\left. +[F^{(2)}(\mu_\tau) + 2F^{(1)}(\mu_\tau)]\mu_\tau^{(1)} + [F^{(1)}(\mu_\tau) + 2F(\mu_\tau)]\mu_\tau^{(2)}\right].
\end{aligned} \tag{C.3}$$

Thus,

$$\begin{aligned}
\widetilde{B}_\tau(x) - B_\tau(x) = &\frac{(2\tau - 1)}{\tau\,[1 - F\,(\mu_\tau)] + (1-\tau)F\,(\mu_\tau)} \\
&\times \left[f'(\mu_\tau)\left(\mu_\tau^{(1)}\right)^3 + [f^{(1)}(\mu_\tau) + 2f(\mu_\tau)]\left(\mu_\tau^{(1)}\right)^2 + 3f(\mu_\tau)\mu_\tau^{(1)}\mu_\tau^{(2)} \right. \\
&\left. +[F^{(2)}(\mu_\tau) + 2F^{(1)}(\mu_\tau)]\mu_\tau^{(1)} + [F^{(1)}(\mu_\tau) + 2F(\mu_\tau)]\mu_\tau^{(2)}\right] \frac{\boldsymbol{e}_1^\top \boldsymbol{S}^{-1} \boldsymbol{c}_1}{3!} h^3.
\end{aligned}$$

It is clear to see that, when $\tau = 0.5$, $\widetilde{B}_\tau(x) = B_\tau(x)$.

References

Bosq, D. (1999). *Nonparametric Statistics for Stochastic Processes: Estimation and Prediction, 2nd ed.* Lecture Notes in Statistics 110. Springer-Verlag, New York.

Fan, J. and Yao, Q. (2003). *Nonlinear Time Series: Nonparametric and Parametric Methods.* Springer.

Hall, P. and Heyde, C. (1980). *Martingale limit theory and its applications.* Academic Press, New York.

Mack, Y. and Silverman, B. (1982). Weak and strong uniform consistency of kernel

regression estimates. *Zeitschrift fur Wahrscheinlichkeitsthorie und Verwandte Gebiete*, 61:405–415.

Masry, E. and Fan, J. (1997). Local polynomial estimation of regression functions for mixing processes. *Scandinavian Journal of Statistics*, 24:165–179.

Stute, W. (1984a). Asymptotic normality of nearest neighbor regression funciton estimates. *The Annals of Statistics*, 12:917–926.

Stute, W. (1984b). The oscillation behavior of empirical processes: the multivariate case. *The Annals of Probability*, 12:361–379.

Volkonskii, V. and Rozanov, Y. (1959). Some limit theorems for random functions. *Theory of Probability and Its Applications*, 4:178–197.
